# On the Discorhabdins Leading to the Aleutianamine Ring System: A One‐Step in Situ Transformation Characterized Through Computational and Experimental Studies and Its Implications on Biosynthesis, Synthesis, and Pharmacology

**DOI:** 10.1002/anie.7864883

**Published:** 2026-02-15

**Authors:** Cody F. Dickinson, Abhay Potluri, Alison M. Bland, Samuel M. Flipse, George S. Hanna, Ryan T. Wagner, Keith D. Robertson, Thai H. Ho, Daniel J. Sprague, Gerald R. Hoff, Robert P. Stone, Marcus A. Tius, Dean J. Tantillo, Mark T. Hamann

**Affiliations:** ^1^ Department of Drug Discovery & Biomedical Sciences Medical University of South Carolina Charleston South Carolina USA; ^2^ Department of Chemistry and Chemical Biology University of California Davis Davis California USA; ^3^ Department of Biochemistry and Molecular Biology Medical University of South Carolina Charleston South Carolina USA; ^4^ Department of Chemistry University of Hawaii at Manoa Honolulu Hawaii USA; ^5^ Department of Public Health Sciences Medical University of South Carolina Charleston South Carolina USA; ^6^ Department of Molecular Pharmacology and Experimental Therapeutics Mayo Clinic Rochester Minnesota USA; ^7^ Division of Hematology and Medical Oncology Hollings Cancer Center Medical University of South Carolina Charleston South Carolina USA; ^8^ Resource Assessment and Conservation Division Alaska Fisheries Science Center National Marine Fisheries Service National Oceanic and Atmospheric Administration Seattle Washington USA; ^9^ Auke Bay Laboratories Alaska Fisheries Science Center National Marine Fisheries Service National Oceanic and Atmospheric Administration Seattle Washington USA

**Keywords:** aleutianamine, density functional theory reaction mechanistic analysis, discorhabdin rearrangement, natural products, pyrroloiminoquinone natural products

## Abstract

We disclose an efficient transformation of the relatively common pyrroloiminoquinone discorhabdin alkaloids from Alaskan *Latrunculia* spp. to the complex ring system of aleutianamine by an acid catalyzed process that mimics the proposed endogenous genesis from 3‐dihydrodienyl discorhabdin precursors. The azepine ring system is formed by a concerted 1,2‐alkyl shift of a proposed thiocarbenium‐iminium dication and this mechanism is supported by extensive density functional theory (DFT) analysis. Additional in vitro biological data further supports the aleutianamines as novel and highly potent inhibitors of multidrug resistant cancer lines exemplified further with cytotoxicity toward chemotherapy resistant chromophobe renal cell carcinoma (IC_50_ = 130 nM). This report provides a key transformation that allows the incorporation of either synthetic or biosynthetic starting materials leading to the generation of the aleutianamine complex ring system in just a single step from the discorhabdin ring system.

## Graphical abstract

The thiocarbenium ion rearrangement of 3‐dihydrodisorhabdin B greatly increases the recovery of aleutianamine from *Latrunculia* spp., a pyrroloiminoquinone natural product with potent activity against drug‐resistant cancers, collected from the deep‐ocean along the Aleutian Islands. This transformation has significant implications in future synthesis/biosynthesis studies and will help provide the material required for efficacy, pharmacokinetics/pharmacodynamics, toxicology, and mechanism of action studies moving forward.

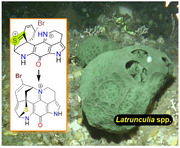

## 1 Introduction

Marine natural products (NP) provide a tremendous level of structural diversity leading to opportunities for the treatment of drug‐resistant human diseases such as cancer [1]. The structural diversity of marine NPs crafted by nature is unmatched in both its creativity, complexity, and bioactivity. Often is the case that the most promising NPs are only available in trace quantities and a comprehensive assessment of their biological potential is thwarted until a reliable, practical supply is established [2]. Depending on the NP origin, the supply issue can only be addressed in a limited number of ways, including semi/total synthesis [3, 4, 5, 6], bacterial culture [2, 7], mariculture or laboratory culture of the macro‐organism [8], or genetic engineering [9]. While these approaches can produce material for biological evaluation, significant amounts of time and resources are devoted to typically access only small quantities to support studies in cell‐based activity assays. Progressing to animal studies, which typically requires at least an order of magnitude increase in production, represents a major hurdle in the drug development process [10]. Positive in vivo results are essential in order to justify the investment in further, but still highly risky, drug development. Thus, practical solutions that produce a reliable NP supply for preclinical development are critical to success.

**SCHEME 1:**
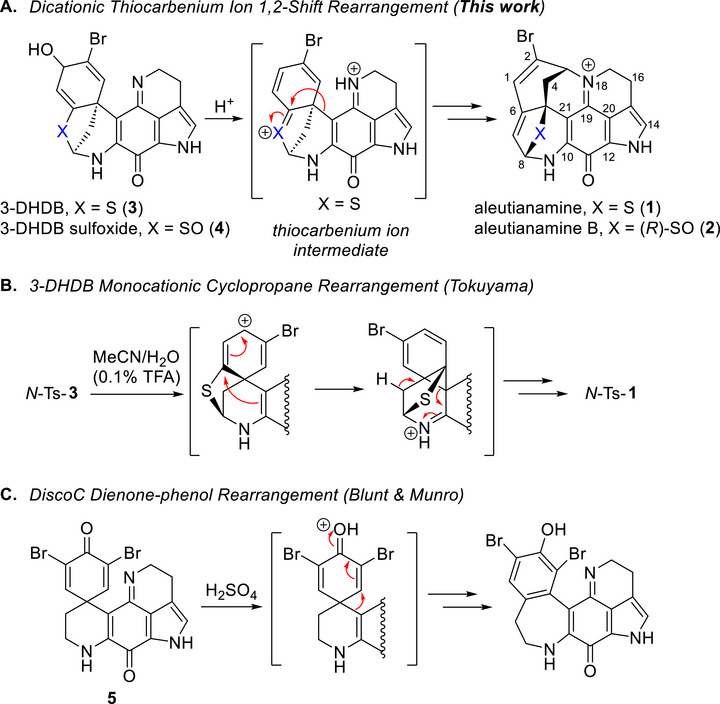
Proposed mechanisms for select discorhabdin rearrangements. (A) This work's proposed dicationic thiocarbenium ion rearrangement of discorhabdin biosynthetic precursors to the aleutianamine ring system. (B) Tokuyama's initially proposed cyclopropane rearrangement of indole *N*‐Ts‐3‐DHDB (*N*‐Ts‐**3**) to *N*‐Ts aleutianamine (*N*‐Ts‐**1**). (C) Blunt & Munro's dienone‐phenol rearrangement of discorhabdin C (DiscoC, **5**).

Polar deep‐ocean marine invertebrates are a source of structurally unique and complex bioactive secondary metabolites. The pyrroloiminoquinone (PIQ) alkaloids, which were the first described in the 1980s and are found in sponge species distributed throughout the world [11, 12, 13, 14, 15, 16], have generated interest due to their complex structures that present challenges in synthesis as well as their potent bioactivity across a variety of diseases and ecological targets [17, 18, 19, 20, 21, 22, 23, 24, 25, 26, 27]. We previously reported the discovery of aleutianamine [28] (**1**) in trace quantities and as a PANC‐1 inhibiting (IC_50_ = 25 nM) 16‐membered macrocyclic PIQ featuring a bridging thioaminal and a vinylic bromide. All of these features are derived from just a tyramine and tryptamine residue with a subsequent addition of both a sulfur and bromine atom. Additionally, we recently reported the (*R*)‐sulfoxide derivative, aleutianamine B (**2**), that has 50‐fold selectivity for ovarian cancer compared to normal cell lines [29]. Herein, we report our continuing investigations of the Alaskan deep‐ocean *Latrunculia* sponges collected as bycatch during the 2024 National Oceanic and Atmospheric Administration's (NOAA) bottom‐trawl survey in the Aleutian Islands of Alaska. We detail the conversion of 3‐dihydrodiscorhabdin B (3‐DHDB, **3**) to aleutianamine that substantially increases the abundance of aleutianamine isolated from these sponges to practical levels for in vivo preclinical assessment. The approach presented here is unique in that it allows for a useful entry point via discorhabdin B that could be applied to alternative production methods (vide supra) useful for generating aleutianamine more effectively. A plausible mechanism involving a 1,2‐shift of a proposed dicationic thiocarbenium ion intermediate for this transformation (Scheme 1A) is detailed and supported by DFT analysis and sheds light on the endogenous synthesis of the aleutianamine ring system. We also report new in vitro data that further supports aleutianamine as a novel nanomolar active multidrug resistant cancer drug lead.

The structure and activity of aleutianamine has been confirmed by others [3, 4, 5, 6]. The strained bromodiene and bridged thioether are the likely pharmacophores responsible in part for its activity [30]. The supply of a molecule with this complexity in order to explore the pharmacology, cell biology, and therapeutic potential is challenging. While numerous total syntheses of aleutianamine [3, 4, 5] have been disclosed, none have produced more than ca. 3 mg [31].

Of interest was the serendipitous discovery of a cationic rearrangement of 3‐DHDB (Scheme 1B) to aleutianamine during HPLC purification of *N*‐Ts‐**3** as initially reported by Tokuyama and later applied by Vanderwal [5, 6]. The rearrangement to the azepine ring system of aleutianamine was initially proposed to occur through a cyclopropane intermediate followed by ring opening triggered by loss of a proton. Related cationic rearrangements of the discorhabdin (Disco) C skeleton in neat sulfuric acid were originally reported by Blunt and Munro and were described as a classical dienone–phenol rearrangement (Scheme 1C) [32]. Yet challenges remain—primarily with the question of process scalability and overall efficiency of the reduction of DiscoB to 3‐DHDB and subsequent cationic rearrangement [6]. However, due to the high potency of aleutianamine it is unlikely that a large quantity will be necessary for preclinical evaluation, thus rapid access to >100 mg would be a significant improvement. A practical solution may entail the semi‐synthetic conversion of abundant biosynthetic precursors from the natural source.

## 2 Results and Discussion

**FIGURE 1:**
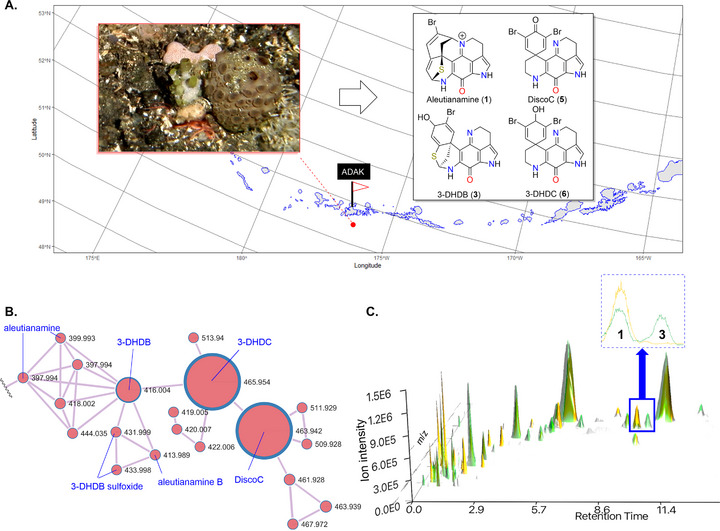
(A) Map of the Aleutian Islands, *x*‐axis = longitude, *y*‐axis = latitude. The collection site of *Latrunculia* spp. south of Adak Island, Alaska is marked. The underwater photo depicts representative examples of *L. oparinae* (left, green specimen) and *hamanni* (right, brown specimen) in life; this photo was taken near Kanaga Island (due west of Adak Island). (B) GNPS analysis of the ethanol extract of *Latrunculia* spp. from Adak Island. The node containing the aleutianamines is annotated. The dot size is correlated to the ion intensity. Task ID = bc76778268d440dbb52ed8401ba621b1. (C) 3D‐graph (x‐axis = retention time (min), y‐axis = *m*/*z*, z‐axis = ion intensity) depicting the pre‐ (green) and post‐acid (yellow) treated ethanol extract. UHPLC‐qTOF chromatogram (dashed blue box) of pre‐ and post‐acid‐treated ethanol extract (20 mg mL^−1^) shows aleutianamine (**1**) and 3‐DHDB (**3**).

Approximately 45 kg of fresh *Latrunculia* spp. (*L. hamanni* and *L. oparinae*) [33] were collected at 36 stations at depths between 86 and 406 m along the Aleutian Archipelago [34]. A preliminary assessment of the PIQ makeup of these sponges was conducted with samples collected at two stations south of Adak Island, Alaska (Figure 1A). The fresh sponge material (8.5 kg) was extracted with ethanol and subjected to GNPS molecular ion networking analysis (Figure 1B), which revealed a large PIQ cluster. DiscoC (**5**) and 3‐dihydrodiscorhabdin C (3‐DHDC, **6**) were identified as the primary PIQs in the extract. Extracted ion analysis of the qTOF‐MS chromatogram for *m*/*z* 397.99 [M]^+^ identified the presence of aleutianamine (Figure S1) in small quantities. Additionally, an *m*/*z* 397.99 fragment ion [M‐H_2_O]^+^ whose parent mass was *m*/*z* 416.00 [M + H]^+^ was detected to which 3‐DHDB (**3**) was assigned. The molecular ion analysis revealed aleutianamine clustered closely with 3‐DHDB. This fragmentation ion lends some support to the hypothesis that aleutianamine may be derived from 3‐DHDB in situ [35]. Aleutianamine B (**2**) was also identified in the cluster (*m*/*z* 413.98) along with the putative 3‐DHDB sulfoxide (*m*/*z* 431.99).

Based on these observations and informed by our prior experience, [28, 36, 37] we subjected the extract to an acid–base partitioning scheme. A total of 35 mg of aleutianamine was isolated as the TFA salt [38] following silica gel fractionation and C_18_ prep‐HPLC purification. This represents the largest production of aleutianamine reported to date [39]. Encouraged by this, we processed the remaining sponge material from the trawl survey [40]. The alkaloid extract (ca. 100 g) was treated with TFA in MeOH/DCM at room temperature. Analysis of the pre‐ and post‐acid‐treated extract showed the complete conversion of 3‐DHDB along with a significant increase of aleutianamine (Figure 1C). A feature‐based molecular network (FBMN) comparison of the pre‐ and post‐acid‐treated extracts revealed the production of additional PIQs that were unique to the acid‐treated extract (Figure S4) [41]. This highlights the acid sensitivity of PIQ NPs and provides a unique approach to access new structures within the PIQ class.

**FIGURE 2:**
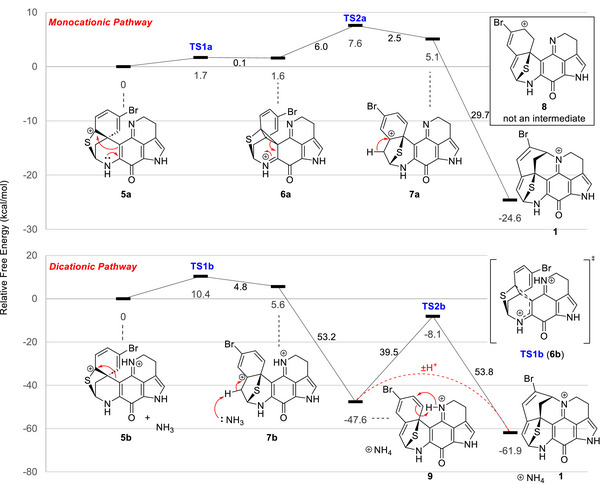
Reaction coordinate diagram of the monocationic (top) and dicationic (bottom) rearrangement of 3‐DHDB to aleutianamine. Relative energies (kcal mol^−1^) of intermediates and transition structures are shown [M062X/6‐311+G(d,p)]. *Note*: While an intramolecular proton transfer of **9** to form **1** was predicted to have a high barrier, it is likely to be a fast process that is mediated by water molecules with the assistance of external acids/bases (red‐dashed curved line).

Previous reports on the acid‐catalyzed rearrangement of the discorhabdins (Scheme 1) have described the process as a dienone–phenol rearrangement or indicated the rearrangement as a monocationic process with a cyclopropane intermediate [5, 6, 32]. We sought to gain insight for this process by addressing two fundamental questions through DFT analysis: 1, does the mechanism involve a concerted 1,2‐alkyl shift or a cyclopropane intermediate, and, 2, is a mono or dicationic [42, 43, 44, 45] rearrangement process taking place? [46] The first question simplifies to one of whether or not there is a barrier for the conversion of a cyclopropane‐like structure to product. The second question arises from the expected basicity of the imine nitrogen atom. Based on our experience, PIQs are isolated and handled as bench stable iminium salts, which makes it unclear whether or not the cationic salt form disfavors carbocation formation necessary for rearrangement. Furthermore, if the salt form does undergo rearrangement, when will this site will be deprotonated to form the N‐bridge? A summary of our findings is illustrated in Figure 2.

A facile rearrangement process in both the mono and dicationic pathways is predicted to occur with overall barriers of ≤10 kcal mol^−1^. A stepwise alkyl shift of the thiocarbenium ion **5a** leading to cyclopropane intermediate **6a** was found in the monocationic pathway; however, a 0.1 kcal mol^−1^ reversion barrier back to precursor **5a** exists. Intermediate **6a** ring opens to carbocation intermediate **7a**. Proton loss is followed directly by reprotonation and cyclization to form aleutianamine. The carbocation **8** is not an intermediate in this pathway. In contrast, the thiocarbenium dication **5b** undergoes a concerted 1,2‐shift to intermediate carbocation **7b** (supported by an intrinsic reaction coordinate [IRC] calculation from **TS1b**). The thiocarbenium ion intermediate **5b** has a strong donor‐acceptor interaction between the sulfur lone pair and the empty *p*‐orbital of the carbocation [46]. Proton loss from intermediate **7b** leads to triene intermediate **9**. A high barrier for an intramolecular proton transfer in intermediate **9** to form aleutianamine was found and suggests that external acids/bases likely mediate this proton transfer. For additional computational results see the Table S3 and Figures S8–S10.

**SCHEME 2:**
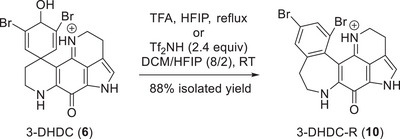
Conversion of 3‐DHDC (**6**) to 3‐DHDC‐R (**10**).

Evidence in support of the dicationic rearrangement pathway is provided by the following. Treating the free base of 3‐DHDC (**6**, orange solution) with 1 equiv. of TFA in hexafluoroisopropanol (HFIP) produced a purple 3‐DHDC TFA salt (Table S1, Figures S2 and S3). Addition of excess TFA and heating at reflux overnight led to a complete reaction that produced the 3‐dihydrodiscorhabdin C rearrangement (3‐DHDC‐R, **10**, Scheme 2, Table S2, Figures S5 and S6) product [47]. In addition, we treated the 3‐DHDC TFA salt with 2.4 equivalents [48] of bis(trifluoromethan)sulfonimide (Tf_2_NH) in a mixture of DCM/HFIP at room temperature. This led to a complete reaction to 3‐DHDC‐R in 88% isolated yield. Dicationic electrophiles from N‐containing precursors are typically produced thermally or with the assistance of superacids [42, 43, 44, 45]. The evidence presented here strongly suggests the salt form undergoes a dicationic rearrangement. Given that 3‐DHDB was originally isolated as a formate salt [37], it is likely the rearrangement process begins from the iminium salt and proceeds through a dicationic mechanism (see Scheme S1).

**FIGURE 3:**
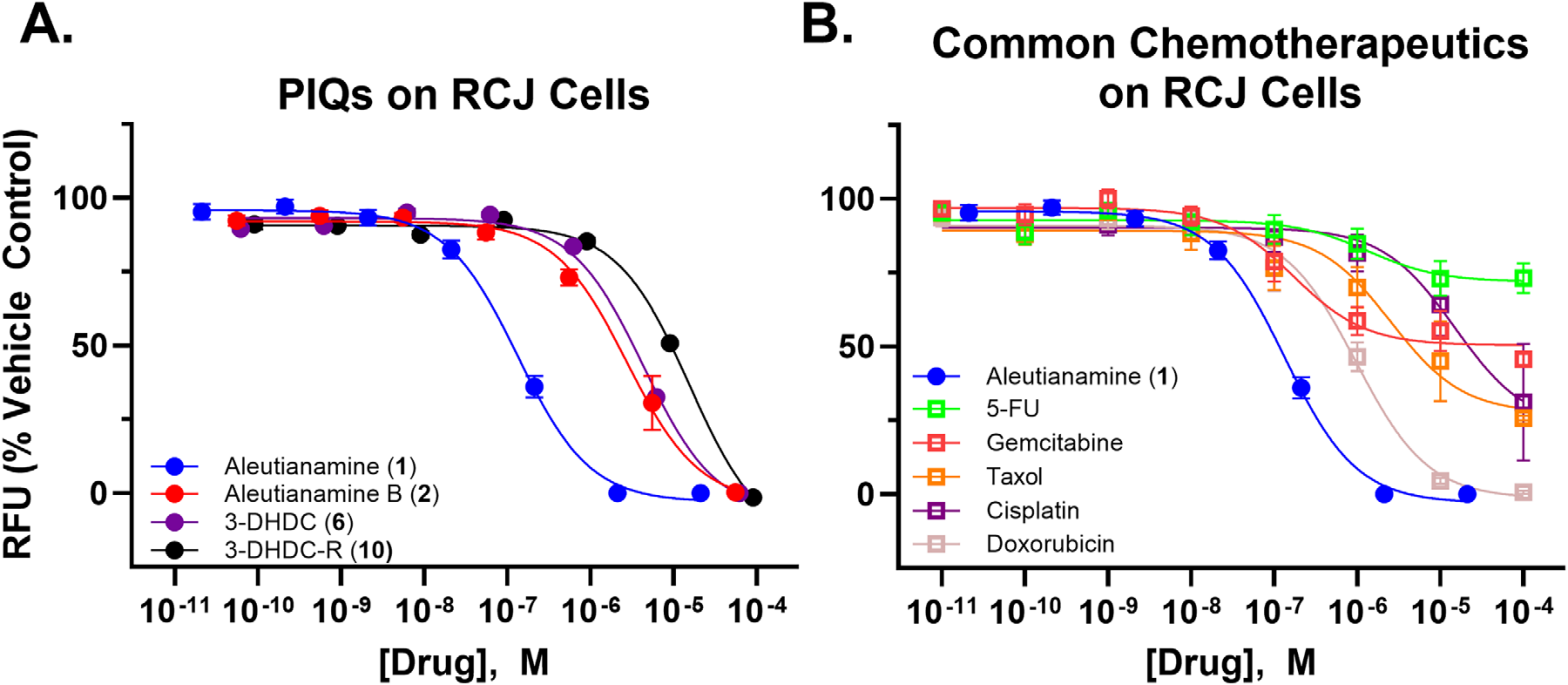
Concentration response curves of aleutianamine (blue circles), aleutianamine B (red circles), 3‐DHDC (purple circles) and 3‐DHDC‐R (black circles) on (A) chromophobe renal cell carcinoma RCJ cells; (B) comparison with common chemotherapeutics. Points are plotted as %RFU of vehicle control (0.1% DMSO in HBSS). Data presented as mean ± SEM of ≥3 biological replicates comprised of technical duplicates.

We tested compounds **1**, **2**, **6**, and **10** for in vitro activity against chromophobe renal cell carcinoma (ChRCC, Figure 3A). ChRCC is a rare type of kidney cancer which is genetically, genomically, and metabolically distinct from other kidney cancers and responds poorly to standard cytotoxic drugs, targeted therapies, and immune checkpoint inhibitors [49]. Our group previously generated a patient derived xenograft (PDX) from a chromophobe renal cell carcinoma that did not respond to targeted therapy and immunotherapy [50, 51]. The derived renal cancer cells, RCJ, were treated with a single dose of compounds **1**, **2**, **6**, and **10** and incubated for five days [52]. RCJ cells treated with aleutianamine and aleutianamine B (Figure 3A) were killed with a single dose with an IC_50_ = 0.13 ± 0.02 µM and IC_50_ = 2.7 ± 0.61 µM, respectively. Both 3‐DHDC (IC_50_ = 4.3 ± 0.35 µM) and 3‐DHDC‐R (IC_50_ = 15.5 ± 1.1 µM) were bioactive, killing the cells in the low micromolar range. This rank order of potency also holds true for 786‐O cells, a common clear cell kidney cancer cell line (Figure S7). The bioactivity of aleutianamine is noteworthy as it also outperformed standard chemotherapeutics on RCJ cells (Figure 3B). For example, 5‐fluorouracil produced a 25% response at 100 µM and gemcitabine showed a ca. 50% response at 100 µM. Taxol demonstrated a ca. 75% response with an IC_50_ ≈ 2.5 µM, and cisplatin exhibited an IC_50 _> 15 µM. Doxorubicin was the only chemotherapeutic examined that completely killed RCJ cells (IC_50_ = 0.96 ± 0.20 µM). This is an order of magnitude less potent than aleutianamine.

In summary, we have demonstrated the rearrangement of the discorhabdins from *Latrunculia* spp., a critical transformation of relatively common PIQs, into the azepine scaffold found in the highly active aleutianamine class. While a reliable yield of the 3‐DHDB transformation has yet to be determined, we estimate that it is an efficient (>85% yield) process based on our assessment of mass spectral data that indicates complete conversion of 3‐DHDB (Figure 1C), the high efficiency of the 3‐DHDC rearrangement, and the mechanistic analysis of the reaction by DFT, which is supportive of a singular pathway to product. This transformation proceeds through a stabilized thiocarbenium ion, a pathway that may be operational in related biosyntheses. Further mechanistic investigations are being carried out by our lab. The preparation of biologically significant PIQs from more abundant discorhabdins demonstrates the value of this transformation. To this end, we have begun studies to transform 100+ g of *Latrunculia* spp.‐derived alkaloids to increase the recovery of aleutianamine and of novel PIQs. While our initial assessment of the NCI *Latrunculia* sponge collection indicated no viable source of aleutianamine [18], we now estimate with the help of this transformation we will recover approximately 150 mg of aleutianamine from *Latrunculia* collected in the Aleutian Islands. We anticipate enough material to assess in vivo efficacy, maximum tolerated dose, pharmacokinetics/pharmacodynamics, and begin mechanism of action studies. The mechanism of action of aleutianamine appears to be unique and the biological data presented here supports the potential of this class as the basis for unique cancer therapeutics. We demonstrate this by showing that a PDX model of ChRCC is susceptible to both discorhabdins and aleutianamines, with aleutianamine showing potency in the nanomolar range.

## Conflicts of Interest

The authors declare no conflicts of interest.

## Supporting information




**Supporting File 1**: The authors have cited additional references within the Supporting Information [28, 41, 53–65].

## Data Availability

The data that support the findings of this study are openly available in ioChem‐BD repository at https://doi.org/10.19061/iochem‐bd‐6‐588. The raw NMR experimental data for aleutianamine, 3‐dihydrodiscorhabdin C, and 3‐dihydrodiscorhabdin C rearrangement product has been deposited in the Natural Products Magnetic Resonance Database (NP‐MRD, www.np‐mrd.org) and is available under the accession numbers NP0352131, NP0352132, and NP0352133, respectively.
