# Supplementary material for: On the Discorhabdins Leading to the Aleutianamine Ring System: A One‐Step in Situ Transformation Characterized Through Computational and Experimental Studies and Its Implications on Biosynthesis, Synthesis, and Pharmacology
Source: Angew Chem Int Ed Engl. 2026 Feb 15;65(17):e7864883. doi: 10.1002/anie.7864883 (PMC13098300; doi:10.1002/anie.7864883)
Supplement: Supplementary file 1 — Supporting File 1: The authors have cited additional references within the Supporting Information [28, 41, 53–65]. [file ANIE-65-e7864883-s001.pdf]

Supporting Information

for

On the Discorhabdins Leading to the Aleutianamine Ring System: A One-Step in Situ Transformation Characterized through Computational and Experimental Studies and its Implications on Biosynthesis, Synthesis and Pharmacology

Cody F. Dickinson,*[a] Abhay Potluri,[b] Alison M. Bland,[c] Samuel M. Flipse,[d] George S. Hanna,[e] Ryan T. Wagner,[f] Keith D. Robertson,[f] Thai H. Ho,[g] Daniel J. Sprague,[c] Gerald R. Hoff,[h] Robert P. Stone,[i] Marcus A. Tius,[d] Dean J. Tantillo,[b] and Mark T. Hamann*[a]

[a] Department of Drug Discovery & Biomedical Sciences, Medical University of South Carolina, Charleston, SC, USA

[b] Department of Chemistry and Chemical Biology, University of California Davis, Davis, CA, USA

[c] Department of Biochemistry and Molecular Biology, Medical University of South Carolina, Charleston, SC, USA

[d] Department of Chemistry, University of Hawaii at Manoa, Honolulu, HI, USA

[e] Department of Public Health Sciences, Medical University of South Carolina, Charleston, SC, USA

[f] Department of Molecular Pharmacology and Experimental Therapeutics, Mayo Clinic, Rochester, MN, USA

[g] Division of Hematology and Medical Oncology, Hollings Cancer Center, Medical University of South Carolina, Charleston, SC, USA

[h] Resource Assessment and Conservation Division, Alaska Fisheries Science Center, National Marine Fisheries Service, National Oceanic and Atmospheric Administration, Seattle, WA, USA

[i] Auke Bay Laboratories, Alaska Fisheries Science Center, National Marine Fisheries Service, National Oceanic and Atmospheric Administration, Seattle, WA, USA

E-mail: hamannm@musc.edu; dickinco@musc.edu

Table of contents:

General Experimental S2

Extraction and Isolation of Pyrroloiminoquinone Alkaloids S3

Rearrangement of 3-Dihydrodiscorhabdin C. S10

Cell-Titer Blue Assay S12

DFT Methods S13

References S15

NMR Spectra S16

General Experimental

Reactions that required heating were carried with a stir-hot plate using a heated external oil bath. Isopropanol (HPLC grade), acetonitrile (HPLC grade), ethanol (anhydrous, USP), and hexanes (ACS grade) were purchased from Fisher and used without further purification. Water was purified through a Milliq purification system. ^{1}H NMR and $^{13}C\{^{1}H\}$ NMR spectra were measured on an Agilent 600 DD2 (600 MHz/150 MHz) or Bruker (600 MHz/150 MHz) spectrometer at ambient temperature. Chemical shifts are reported in parts per million (ppm) and are referenced to the solvent (*e.g.,* δ 7.26 for $CHCl_3$; δ 77.0 for $CDCl_3$). Multiplicities are indicated as follows: br (broadened), s (singlet), d (doublet), t (triplet), q (quartet), pent (pentet), sext (sextet), sept (septet), etc. or m (multiplet). Coupling constants (*J*) are reported in Hertz (Hz). High performance liquid chromatography (HPLC) analyses and purification were performed using a Waters instrument using UV detection at 254 nm. High-resolution mass spectra (HRMS) were obtained with a Bruker qTOF and quaternary Elute system. Thin layer chromatography (TLC) was performed on glass plates, 250 µm, particle size 5–17 µm, pore size 60 Å. All reactions were monitored by TLC and analyzed under UV (254 and/or 365 nm) light and visualized using either PAA or KMnO4 stains. Silica gel flash column chromatography was performed on silica gel, 200–400 mesh or premium silica gel, 60 Å, 40–75 µm. Purity and homogeneity of all materials was determined by TLC, ^{1}H NMR, $^{13}C\{^{1}H\}$ NMR, LCMS, and qNMR. The NMR experimental data for aleutianamine, 3-dihydrodiscorhabdin C, and 3-dihydrodiscorhabdin C rearrangement product has been deposited in the Natural Products Magnetic Resonance Database (NP-MRD, www.np-mrd.org) and is available under the accession numbers NP0352131, NP0352132, and NP0352133, respectively.

Extraction and Isolation of Pyrroloiminoquinone Alkaloids.

Latrunculia spp. were collected off shore of Adak, AK during NOAA's 2024 biannual bottom trawl survey of the Aleutian Islands. The sponges were recovered following a trawl at depths of 150–180 m with bottom surface temperatures approximately 6–7 °C. A mixture of wet, torn *L. oparinae* and *hamanni* were extracted by soaking with 200 proof ethanol (x3) for 24-36 hr. The extract was decanted and filtered through Celite and concentrated to give ca. 350 g of a sticky tar. A small aliquot (ca. 25 mg) of the crude ethanol extract was passed through a short C_{18} cartridge with methanol and analyzed by qTOF-LCMS (below, linear ramp of 2% to 12% IPA/H_2O w/ 0.1% TFA over 15 min) and GNPS molecular ion networking.

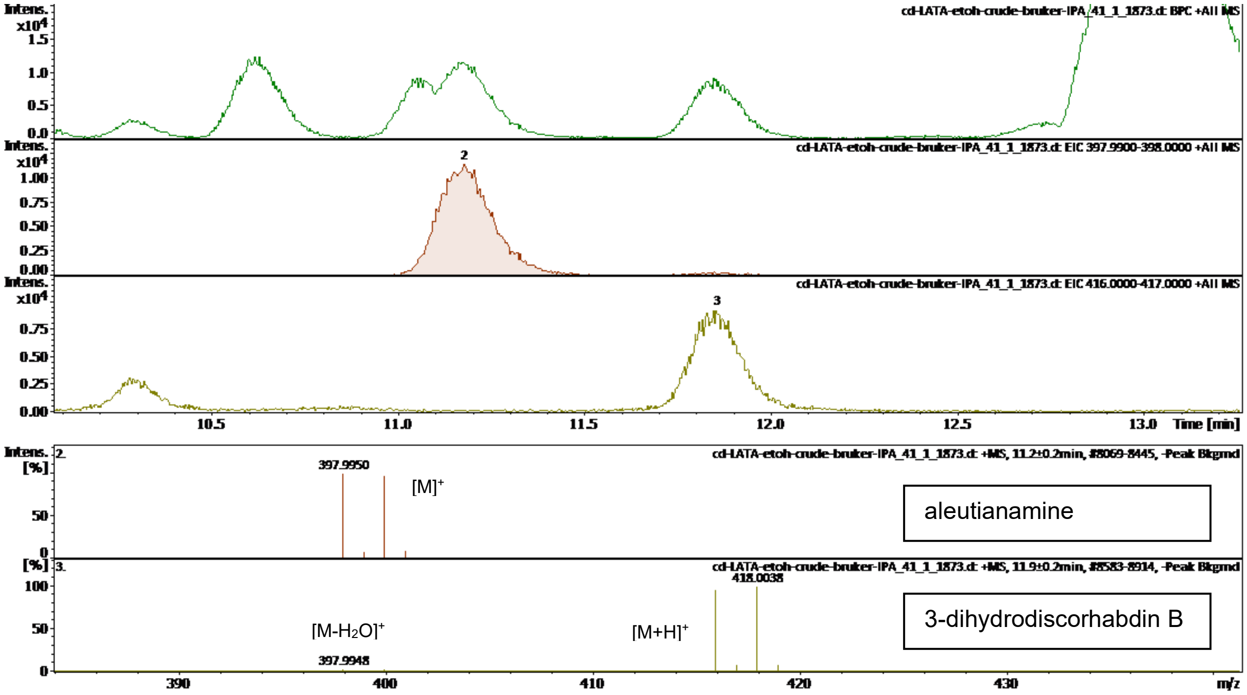

The crude ethanol extract was defatted by partitioning between 6 M HCl and hexanes and washing the aqueous phase with hexanes (x3). The acidic aqueous phase was basified with solid $NaHCO_3$, extracted with 25% IPA/DCM (x5), and dried over anhydrous sodium sulfate. After decanting and filtering of the drying agent, the organic extracts were acidified with TFA and concentrated to dryness to give ca. 25 g of total alkaloids. The crude alkaloid extract was dry loaded onto silica gel and eluted with DCM then 5% -> 10% -> 20% -> 100% MeOH/DCM doped

with ca. 1% TFA. Approximately 3-5 column volumes of eluent were passed through per step and approximately 50 mL fractions were collected and combined (TLC guided). This amounted to a total of seven fractions. F1, 439 mg, F2, 303 mg, F3, 3.8 g, F4, 2.7 g, F5, 7.9 g, F6, 7.5 g, and F7, 2.3 g. These silica gel fractions were analyzed by qTOF-LCMS for m/z 397.99. Fractions F2 and F3 were found to contain aleutianamine. These fractions were then subjected to reversed phased prep-HPLC using a Kinetex C_{18} 250x21 mm, 15 mL/min with an elution program of: 2% MeCN/H_2O (0.1% TFA) equilibration, then isocratic 5% MeCN/H_2O (0.1% TFA) for 15 min, isocratic 10% MeCN/H_2O (0.1% TFA) for 15 min, then isocratic 15% MeCN/H_2O (0.1% TFA) for 20 min, then a linear ramp gradient to 35% MeCN/H_2O (0.1% TFA) over 20 min. The fractions containing aleutianamine were identified by LCMS analysis. Further purification was achieved by reversed phase prep-HPLC using a Kinetex C_{18} 250x21 mm, 20 mL/min with a linear ramp gradient from 2% to 15% IPA/H_2O (0.1% TFA) over 35 min. Aleutianamine was isolated as a hygroscopic TFA salt hydrate (22% wt aleutianamine by qNMR) as an olive-green film (35 mg). Spectral data matched those previously reported in the literature.[28]

^{1}H NMR (600 MHz, methanol-d_4) δ 7.14 (s, 1H), 7.08 (d, J = 1.0 Hz, 1H), 5.97 (d, J = 3.4 Hz, 1H), 5.41 (d, J = 3.4 Hz, 1H), 5.12 (t, J = 3.0 Hz, 1H), 4.26 (dd, J = 10.5, 4.3 Hz, 1H), 4.24 (dd, J = 10.5, 4.3 Hz, 1H), 3.26 – 3.20 (m, 1H), 3.09 (ddd, J = 16.4, 4.3, 3.4 Hz, 1H), 2.66 (dd, J = 12.8, 3.0 Hz, 1H), 2.56 (dd, J = 12.8, 3.0 Hz, 1H); ^{13}C NMR (150 MHz, methanol-d_4) δ 168.6, 149.8, 142.9, 142.2, 129.2, 126.6, 125.7, 122.6, 119.3, 118.1, 112.5, 101.8, 65.4, 64.9, 54.0, 32.5, 21.2. qTOF-LCMS trace is shown in **Figure S1**.

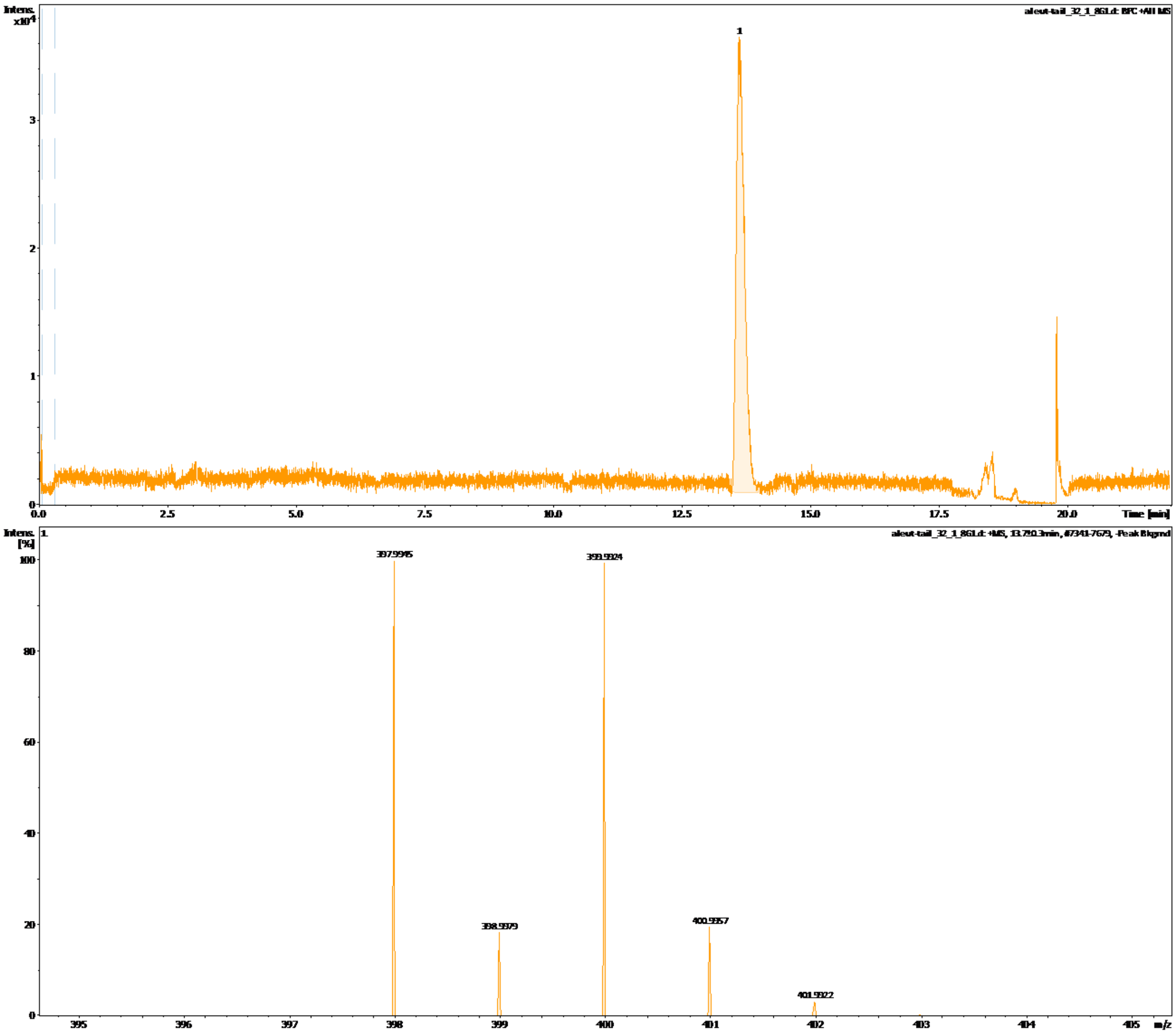

Figure S1: qTOF-LCMS trace of aleutianamine (**1**).

A plausible mechanism for the rearrangement of 3-DHDB to aleutianamine is summarized in **Scheme S1**. Loss of water from the salt form of 3-DHDB leads to a stabilized thiocarbenium ion intermediate **5b** which undergoes a concerted 1,2-alkyl shift (TS structure **6b**) to provide carbocation intermediate **7b** that rapidly loses a proton to form triene **9**. In the case of 3-DHDB, the sulfur atom likely assists in the rate of formation of the cation by stabilization of the intermediate. Compound **9** undergoes a series of proton transfers that leads to a concerted ring closure of triene **11** to form the quaternary iminium-bridge. This last step is most likely a rapid process assisted by solvent *ex vivo* or is enzyme catalyzed within the sponge.

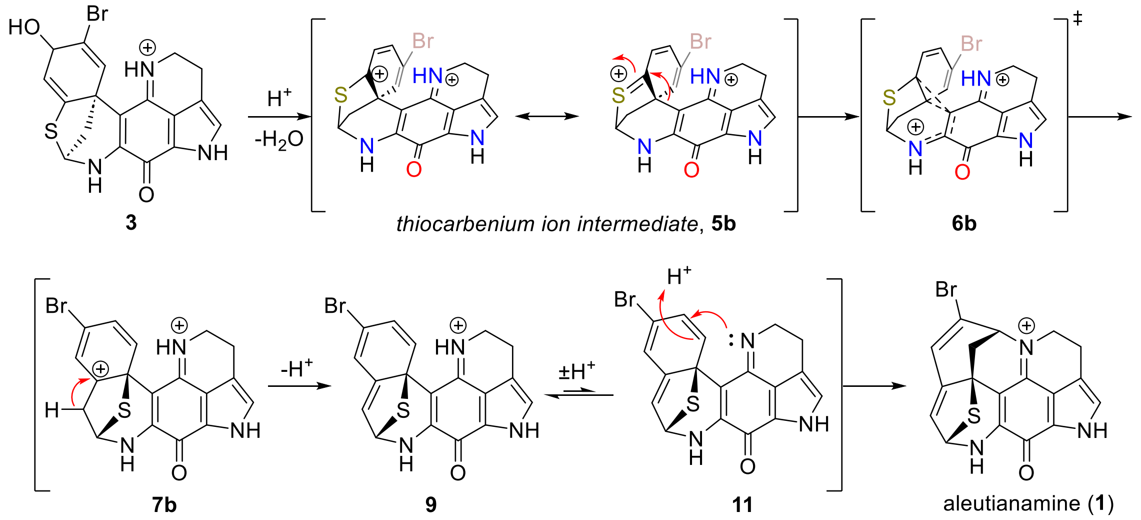

Scheme S1. Postulated mechanism for the transformation of 3-DHDB to aleutianamine.

3-Dihydrodiscorhabdin C (3-DHDC) was found in the same fractions as aleutianamine and was separable from aleutianamine on reversed phase using IPA/H_2O mixtures as eluent, as described above. A total of 395 mg of the TFA salt was isolated as a dark purple solid. NMR data in methanol-d_4 is listed in **Table S1** and 2D NMR correlation maps are shown in **Figure S2**. A qTOF-LCMS trace is shown in **Figure S3**. Note: 3-DHDC is hygroscopic.

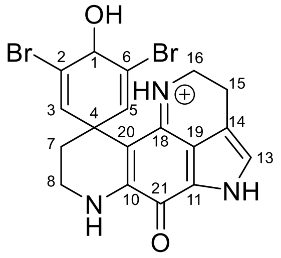

3-dihydrodiscorhabdin C (3-DHDC, **6**)

Atom	^{1}H (600 MHz) δ ppm	^{13}C (150 MHz) δ ppm
1	4.70 (s, 1H)	71.8
2, 6	-	123.7
3, 5	6.44 (s, 2H)	135.1
4	-	43.6
7	1.95 (t, *J* = 5.7 Hz, 2H)	35.2
8	3.65 (t, *J* = 5.7 Hz, 2H)	38.6
10	-	153.8
11	-	124.7
13	7.15 (s, 1H)	127.7
14	-	120.9
15	2.89 (t, *J* = 7.5 Hz, 2H)	19.2
16	3.78 (t, *J* = 7.5 Hz, 2H)	44.2
18	-	155.8
19	-	125.1
20	-	94.1
21	-	166.6

Table S1. ^{1}H and ^{13}C{^{1}H} NMR data in methanol-d_4 for 3-DHDC.

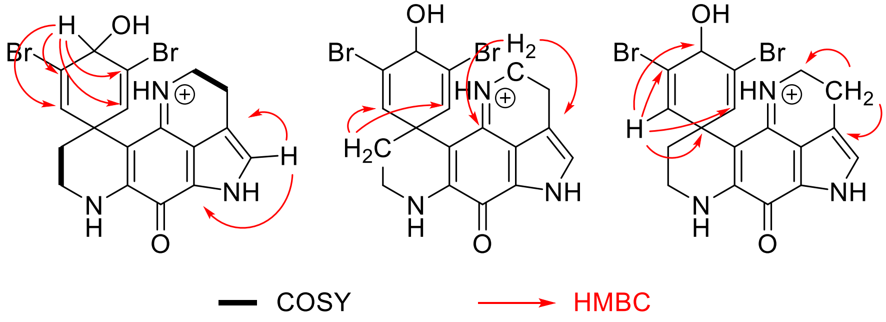

Figure S2. Correlation maps of 3-DHDC.

HRMS: calcd for $[M+H]^+$ $C_{18}H_{16}Br_2N_3O_2^+$ m/z 463.9604; found: 463.9602.

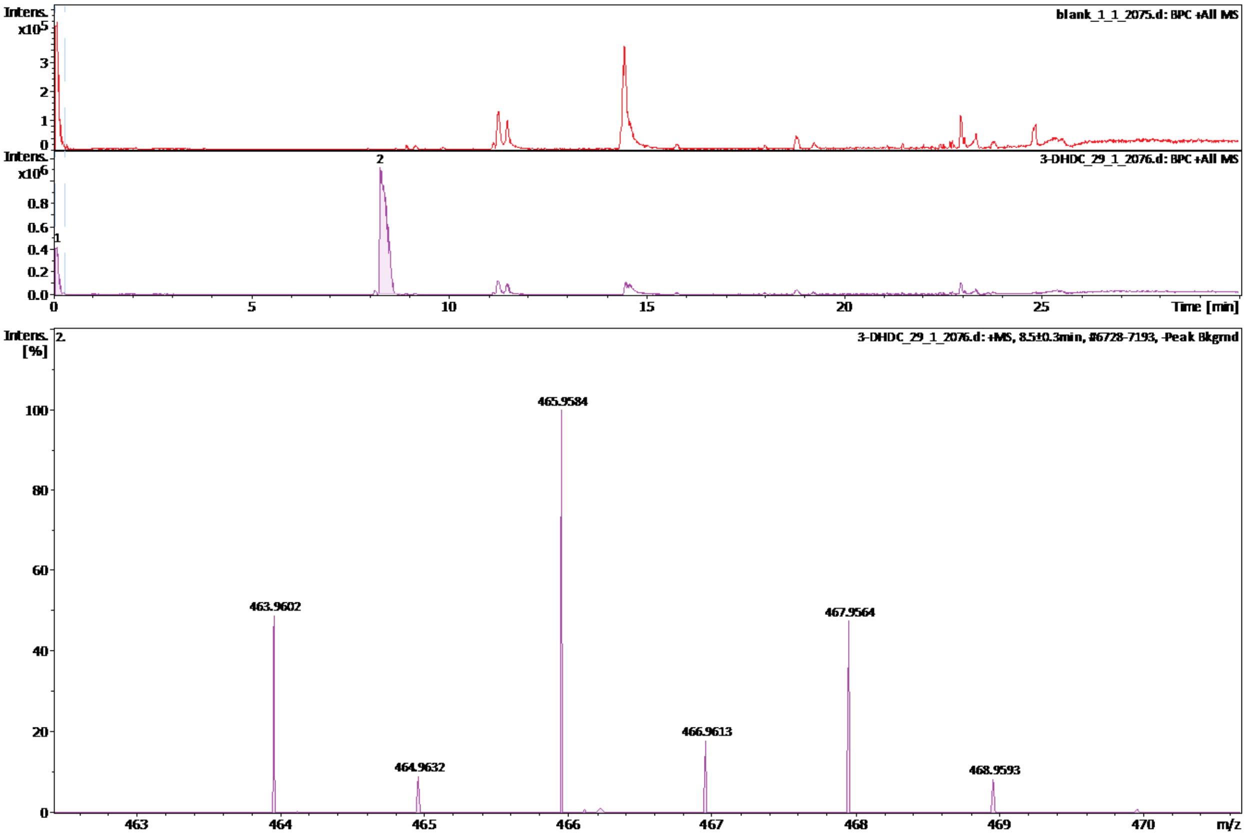

Figure S3. qTOF-LCMS trace of 3-DHDC (bottom) and a blank (top).

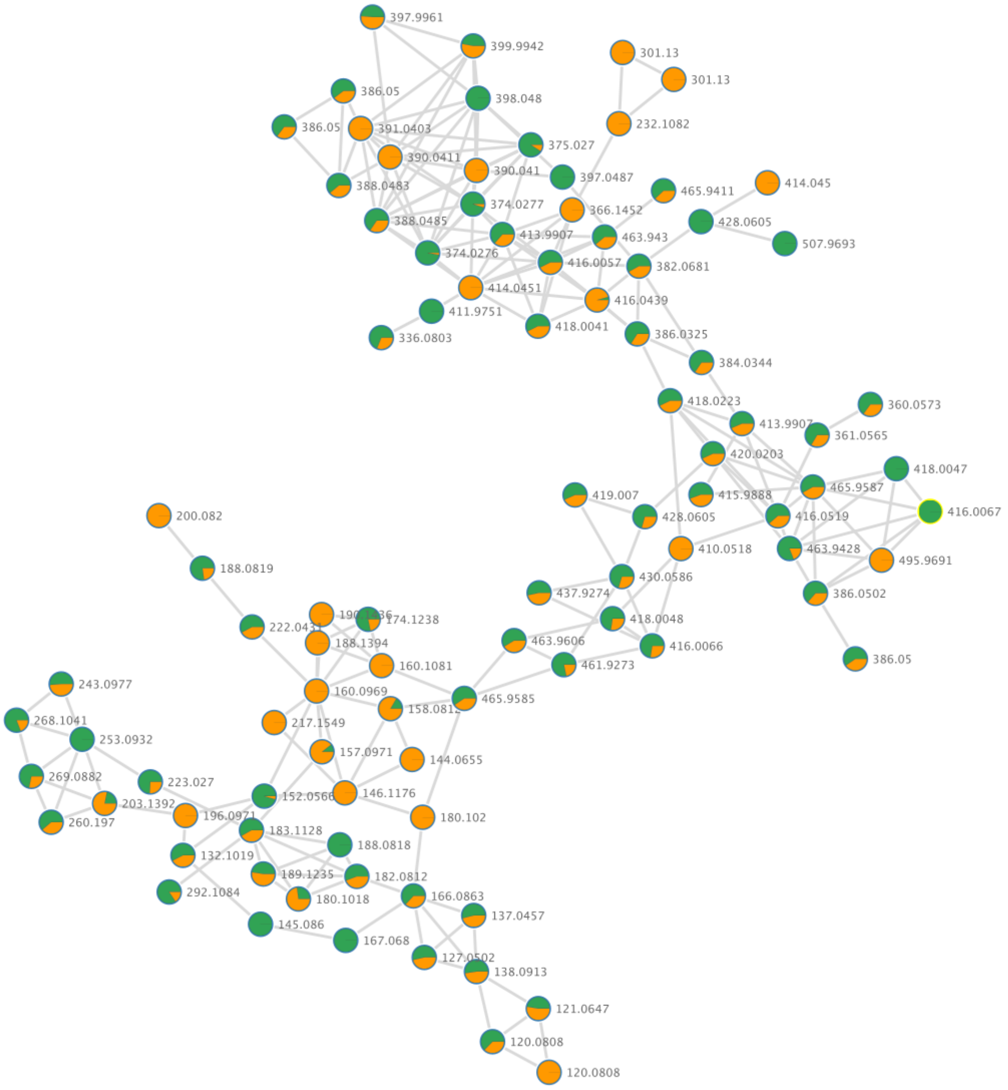

Figure S4. Feature Based Molecular Network (top) of the discorhabdin-cluster from the *Latrunculia* crude extract before (green) and after (orange) treatment with acid. A feature list was generated using MZmine, FBMN files were exported and processed using the “feature_based_molecular_networking_workflow” via GNPS2. Due to sparse fragmentation characteristics of some discorhabdin-related molecules Network_min_cosine was set to 0.4 and networking_min_matched_peaks was set to 4. For all other parameters, defaults were used.[41] (Task ID: b9e2fc772b0645cca1ad4aea302bc1eb) The network was visualized using Cytoscape.

Rearrangement of 3-Dihydrodiscorhabdin C.

To a solution of 3-dihydrodiscorhabdin C TFA salt (62 mg 39% purity wt% by qNMR, 41.7 µmol, 1.0 eq) was dissolved in 20% HFIP in DCM (10 mL) was added Tf_2NH (28 mg, 100 µmol, 2.4 eq). The reaction mixture was stirred at room temperature until completion of the reaction. The reaction mixture was quenched by the addition of sat. aq. sodium bicarbonate and extracted with DCM (x3). The combined organic extracts were washed with brine and dried over anhydrous sodium sulfate. After filtration, TFA was added to the DCM extract and concentrated to dryness. 3-DHDC-R was isolated as the TFA salt by reversed phase prep-HPLC (Kinetex C_{18} 250x21 mm, 254 nm, 20 mL/min, linear ramp over 30 min of 2-35% MeCN/H_2O with 0.1% TFA) as a blue-grey solid that forms blue colored solutions in alcohol (23 mg, qNMR 90% purity, 36.9 µmol, 88% yield). NMR data in methanol-d_4 is listed in **Table S2** and 2D-correlation maps are shown in **Figure S5**. A qTOF-LCMS trace is shown in **Figure S6**.

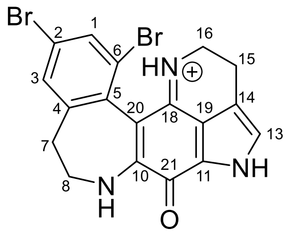

3-dihydrodiscorhabdin C rearranged (3-DHDC-R, **10**)

Atom	^1H (600 MHz) δ ppm	^{13}C (150 MHz) δ ppm
1	7.83 (d, *J* = 2.1 Hz, 1H)	136.1
2	-	123.7
3	7.50 (d, *J* = 2.1 Hz, 1H)	131.8
4	-	147.7
5	-	131.4
6	-	126.5
7a	3.33 (dd, *J* = 14.4, 10.9 Hz, 1H)	34.3
7b	3.05 (dd, *J* = 14.4, 4.3 Hz, 1H)	
8a	4.04 (dd, *J* = 15.3, 4.3 Hz, 1H)	51.8
8b	3.47 (dd, *J* = 15.3, 10.9 Hz, 1H)	
10	-	152.8
11	-	124.1
13	7.24 (s, 1H)	127.7
14	-	121.0
15	3.00 (dd, *J* = 8.9, 6.3 Hz, 2H)	19.5
16a	3.94 (dt, *J* = 14.3, 6.3 Hz, 1H)	44.6
16b	3.76 (dt, *J* = 14.3, 8.9 Hz, 1H)	
18	-	157.7
19	-	125.0
20	-	97.7
21	-	168.4

Table S2. ^1H and $^{13}C\{^1H\}$ NMR data in methanol-d_4 for 3-DHDC-R.

HRMS: calcd for $[M+H]^+$ $C_{18}H_{14}Br_2N_3O^+$ m/z 445.9498; found: 445.9498.

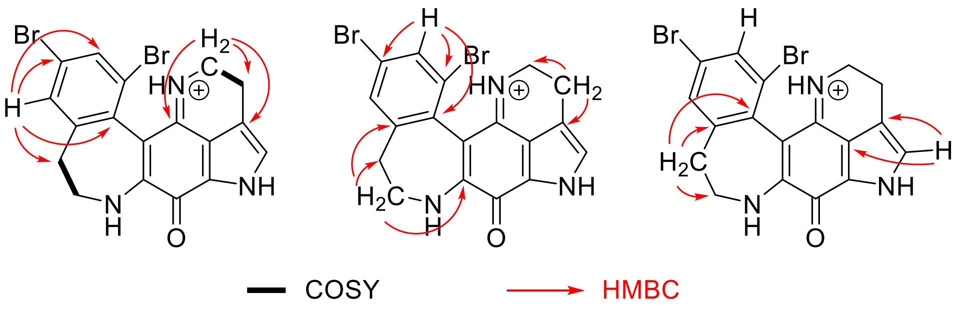

Figure S5. Correlation maps of 3-DHDC-R.

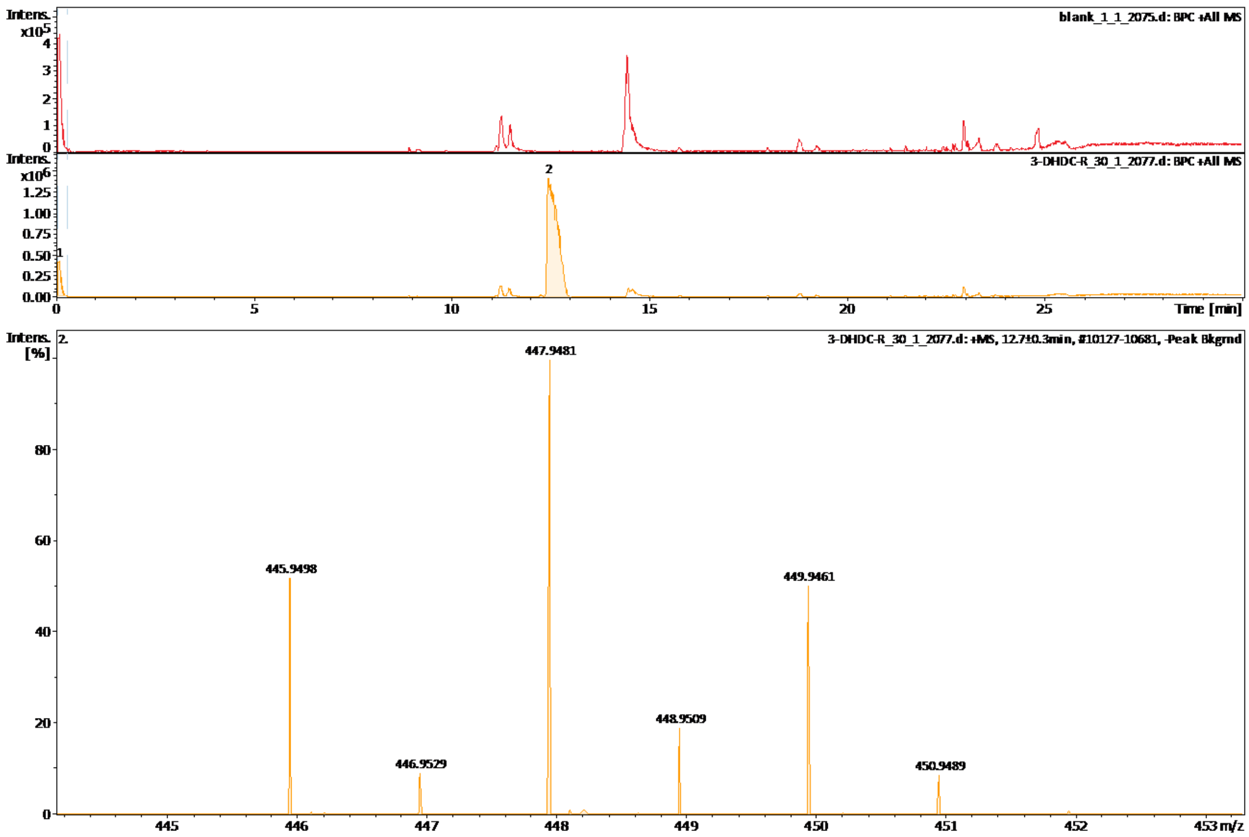

Figure S6. qTOF-LCMS trace of 3-DHDC-R (bottom) and a blank (top).

Cell-Titer Blue Assay

Drug dilutions were prepared at a 10x concentration in drug buffer (Hanks balanced salt solution with Ca^{2+}, Mg^{2+}, 20 mM HEPES, 0.1% BSA, and 0.03% ascorbic acid). The Cell-Titer Blue Assay (Promega) was performed in black-walled, clear-bottomed 96-well plates. In brief, cells were seeded (10,000 cells/well) in complete media (90 µL; For RCJ cells: DMEM, no pyruvate + 5% FBS + 5% GlutaMax; For 786-O cells: RPMI-1640 + 10% FBS), and allowed to adhere to the plate in the incubator for 24 h. Then, 10 µL of drug solution was added, and the cells were incubated for 5 days at 37 °C/5% CO2. Cell-Titer Blue dye (20 µL) was added, and the cells were incubated for 4 h, before fluorescence was read on a Promega GloMax Discover Plate Reader using the default Cell-Titer Blue protocol. Changes in fluorescence are represented as relative fluorescence units (%RFU), and are plotted as %RFU of vehicle control (0.1% DMSO) after subtracting background (positive kill control, 20% DMSO). Concentration-response analysis was performed using sigmoidal curve fitting functions in GraphPad Prism.

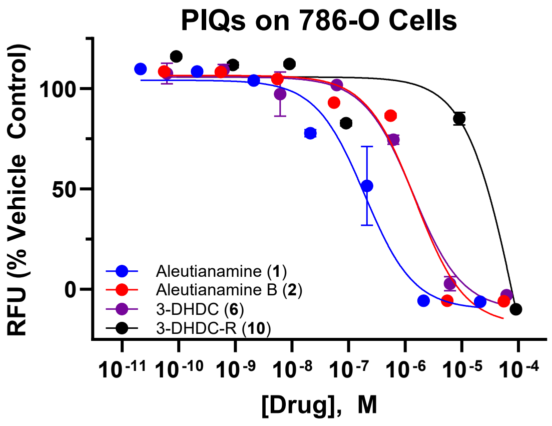

Figure S7. **In vitro cell viability assay.** Concentration response curves of aleutianamine (blue circles), aleutianamine B (red circles), 3-DHDC (purple circles) and 3-DHDC-R (black circles) on clear cell renal cell carcinoma 786-O cells.

DFT Methods

To optimize minima and transition structures, DFT calculations were performed using the M06-2X/6-311+G(d,p) level of theory.[53-56] as implemented in Gaussian 16.C.01.[57] Paton's and Funes-Ardoiz's GoodVibes program was used to employ the roto-harmonic approximation to make thermal corrections to energies.[58] Natural Bond Orbital (NBO) analysis[59, 60] was performed (at the M06-2X/6-311G(d,p) level of theory) to quantify and visualize (via IQMol[61]) orbital interactions in intermediates of interest. Connections between transition structures and minima were confirmed via the intrinsic reaction coordinate (IRC) approach.[62, 63]

Sulfur Lone Pair and Carbocation Interaction:

To better understand the role of the sulfur bridge and whether the lone pair supports the carbocation formation two different sets of analysis were performed. The first was an NBO analysis of compounds **5a** and **5b** (**Table S3** and **Figure S8**). In the case of the dicationic system (**5b**), resonance was present and the system was treated as sulfur-carbocation as a π-bond whereas in the monocationic system (**5a**) the system was treated with a lone pair on the sulfur atom interacting with the carbocation. In both cases, there is a strong interaction energy associated with the sulfur atom and the carbocation.

Donor-Acceptor Interaction	Interaction energy (kcal/mol)
Compound **5a:** $LP_S \leftrightarrow LP^*_{C+}$	139.85
Compound **5b:** $\pi_{(C\text{-}C)} \leftrightarrow \pi^*_{(S\text{-}\mathbf{C})}$	60.73

Table S3: Donor-acceptor interactions quantified via NBO calculations.

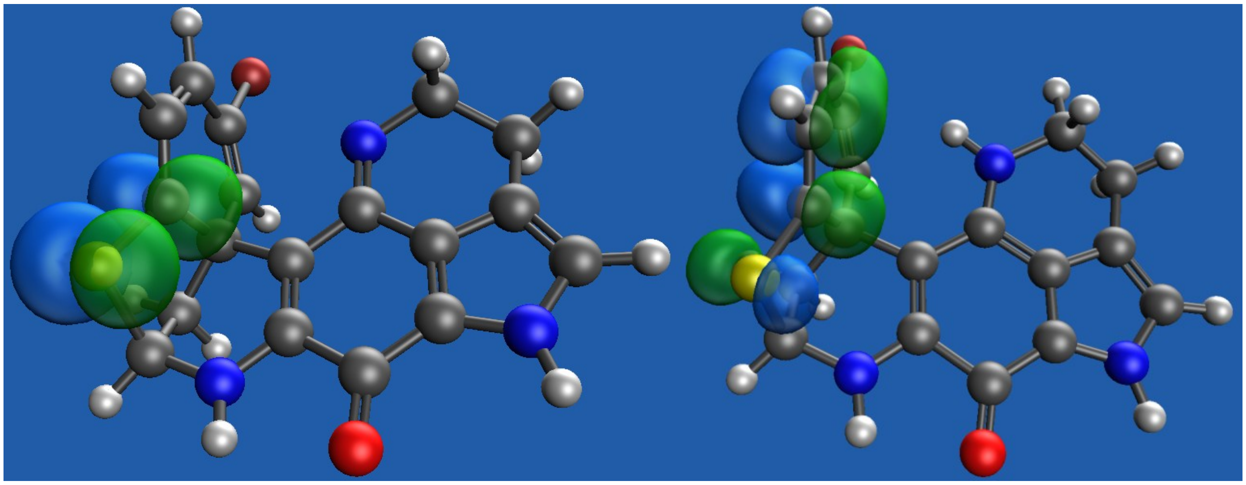

Figure S8: IQMol figures depicting Lewis-type orbitals of donor-acceptor interactions. Left: compound **5a**, monocationic system. Right: compound **5b**, dicationic system.

Our second analysis assessed the behavior of desthio-3-DHDB (free base), which lacks the sulfur atom in comparison to 3-DHDB. Our analysis shows that the carbocation no longer exists as an intermediate along reaction pathways and is only identified in transition states (**Figure S9**). The avoidance of the formation of a discrete carbocation results in the cyclopropyl iminium ion **CmpS1**. This consequently leads to favored reactivity at C-6 with the formation of a new 5-

membered ring with the iminoquinone imine N atom to compound **CmpS2**. Similarly, a pathway was identified where **CmpS1** leads to **CmpS3**.

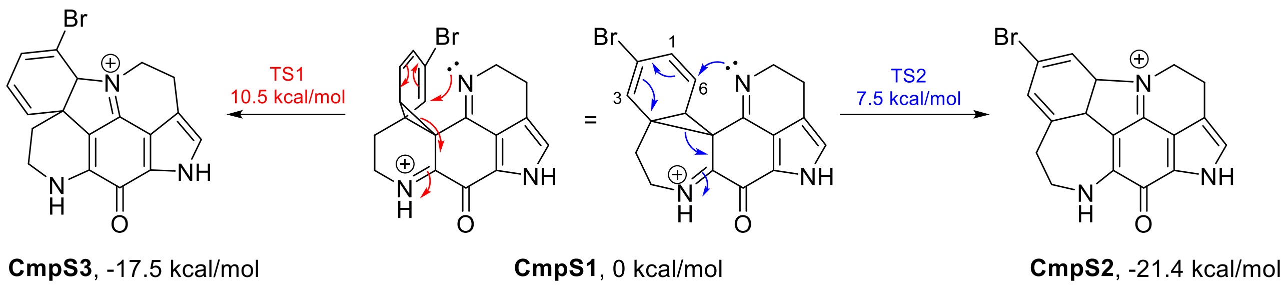

Figure S9. Key structures and their relative energies (kcal/mol) identified for rearrangement pathway for desthio-3-DHDB (no sulfur atom / sulfur bridge).

3-Dihydrodiscorhabdin C (3-DHDC) Rearrangement Mechanism and Discussion:

Mechanistic reaction analysis of the cationic rearrangement of the salt form of 3-DHDC with sulfuric acid is shown in **Figure S10.** Following ionization of the alcohol, **CmpS4** leads to cyclopropyl iminium ion **CmpS5**. Cyclopropane ring opening to give carbocation **CmpS6** followed by rapid proton loss to regenerate aromaticity gives the 3-dihydrodiscorhabdin C rearranged (3-DHDC-R, **10**). Due to the net loss of a proton after **CmpS6**, water was used as a counter ion. The combination of neutralizing the carbocation and reforming aromaticity leads to a significant drop in free energy.

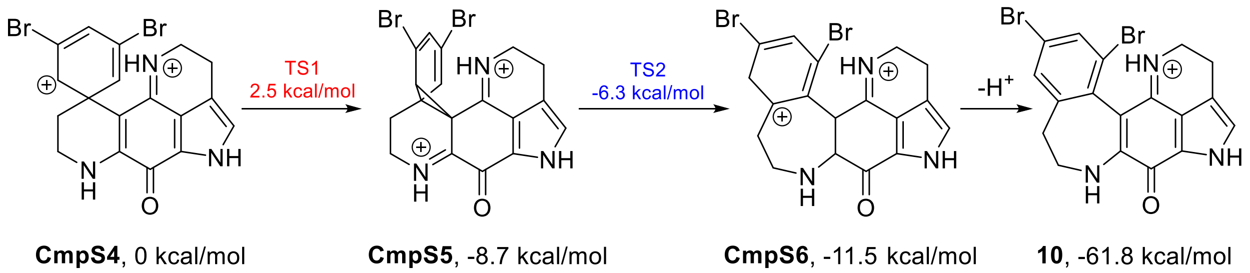

Figure S10. Key structures and their relative energies (kcal/mol) identified for the rearrangement of 3-dihydrodiscorhabdin C to 3-didihydrodiscorhabdin C-rearranged (**10**).

A data set collection of computational results is available in the ioChem-BD repository[64, 65] and can be accessed via https://doi.org/10.19061/iochem-bd-6-588

References

(53) Y. Zhao and D. G. Truhlar. "Density Functionals with Broad Applicability in Chemistry." *Accounts of Chemical Research* 41, no. 2 (2008): 157–167, https://doi.org/10.1021/ar700111a.

(54) Y. Zhao and D. G. Truhlar. "The M06 suite of density functionals for main group thermochemistry, thermochemical kinetics, noncovalent interactions, excited states, and transition elements: two new functionals and systematic testing of four M06-class functionals and 12 other functionals." *Theoretical Chemistry Accounts* 120, no. 1-3 (2007): 215-241, https://doi.org/10.1007/s00214-007-0310-x.

(55) R. Krishnan, J. S. Binkley, R. Seeger, and J. A. Pople, "Self-Consistent Molecular Orbital Methods. XX. A Basis Set for Correlated Wave Functions," *Journal of Chemical Physics* 72, no. 1 (1980): 650–654, https://doi.org/10.1021/ar700111a.

(56) D. McLean and G. S. Chandler, "Contracted Gaussian Basis Sets for Molecular Calculations. I. Second Row Atoms, *Z* =11–18," *Journal of Chemical Physics* 72, no. 10 (1980): 5639–5648, https://doi.org/10.1063/1.438980.

(57) Gaussian 16, Revision C.01, M. J. Frisch, G. W. Trucks, H. B. Schlegel, G. E. Scuseria, M. A. Robb, J. R. Cheeseman, G. Scalmani, V. Barone, G. A. Petersson, H. Nakatsuji, X. Li, M. Caricato, A. V. Marenich, J. Bloino, B. G. Janesko, R. Gomperts, B. Mennucci, H. P. Hratchian, J. V. Ortiz, A. F. Izmaylov, J. L. Sonnenberg, D. Williams-Young, F. Ding, F. Lipparini, F. Egidi, J. Goings, B. Peng, A. Petrone, T. Henderson, D. Ranasinghe, V. G. Zakrzewski, J. Gao, N. Rega, G. Zheng, W. Liang, M. Hada, M. Ehara, K. Toyota, R. Fukuda, J. Hasegawa, M. Ishida, T. Nakajima, Y. Honda, O. Kitao, H. Nakai, T. Vreven, K. Throssell, J. A. Montgomery, Jr., J. E. Peralta, F. Ogliaro, M. J. Bearpark, J. J. Heyd, E. N. Brothers, K. N. Kudin, V. N. Staroverov, T. A. Keith, R. Kobayashi, J. Normand, K. Raghavachari, A. P. Rendell, J. C. Burant, S. S. Iyengar, J. Tomasi, M. Cossi, J. M. Millam, M. Klene, C. Adamo, R. Cammi, J. W. Ochterski, R. L. Martin, K. Morokuma, O. Farkas, J. B. Foresman, and D. J. Fox, Gaussian, Inc., Wallingford CT, 2016.

(58) G. Luchini, J. Alegre-Requena, I. Funes-Ardoiz, and R. Paton, "GoodVibes: Automated Thermochemistry for Heterogeneous Computational Chemistry Data [version 1; peer review: 2 approved With reservations]," *F1000Research* 9 (2020): 291.

(59) F. Weinhold, C. R. Landis, and E. D. Glendening, "What Is NBO Analysis and How Is It Useful?," *International Reviews in Physical Chemistry* 35, no. 3 (2016): 399–440, https://doi.org/10.1080/0144235X.2016.1192262.

(60) Glendening, E.D., Reed, A.E., Carpenter, J.E. and Weinhold, F. (**2003**) NBO Version 3.1. Gaussian Inc., Pittsburgh

(61) http://iqmol.org/ (accessed 2024-03-05).

(62) C. Gonzalez and H. B. Schlegel, "Reaction Path Following in Mass-Weighted Internal Coordinates," *Journal of Physical Chemistry* 94, no. 14 (1990): 5523–5527, https://doi.org/10.1021/j100377a021.

(63) K. Fukui, "The Path of Chemical Reactions—the IRC Approach," *Accounts of Chemical Research* 14, no. 12 (1981): 363–368, https://doi.org/10.1021/ar00072a001.

(64) C. Bo, F. Maseras, and N. López, "The Role of Computational Results Databases in Accelerating the Discovery of Catalysts," *Nature Catalysis* 1, no. 11 (2018): 809–810, https://doi.org/10.1038/s41929-018-0176-4.

(65) M. Álvarez-Moreno, C. de Graaf, N. López, F. Maseras, J. M. Poblet, and C. Bo, "Managing the Computational Chemistry Big Data Problem: The ioChem-BD Platform," *Journal of Chemical Information and Modeling* 55, no. 1 (2015): 95–103, https://doi.org/10.1021/ci500593j.

NMR Spectra

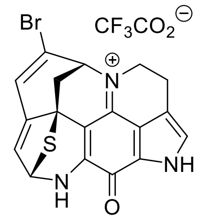

Aleutianamine (**1**)
^{1}H NMR (600 MHz, methanol-d_4)

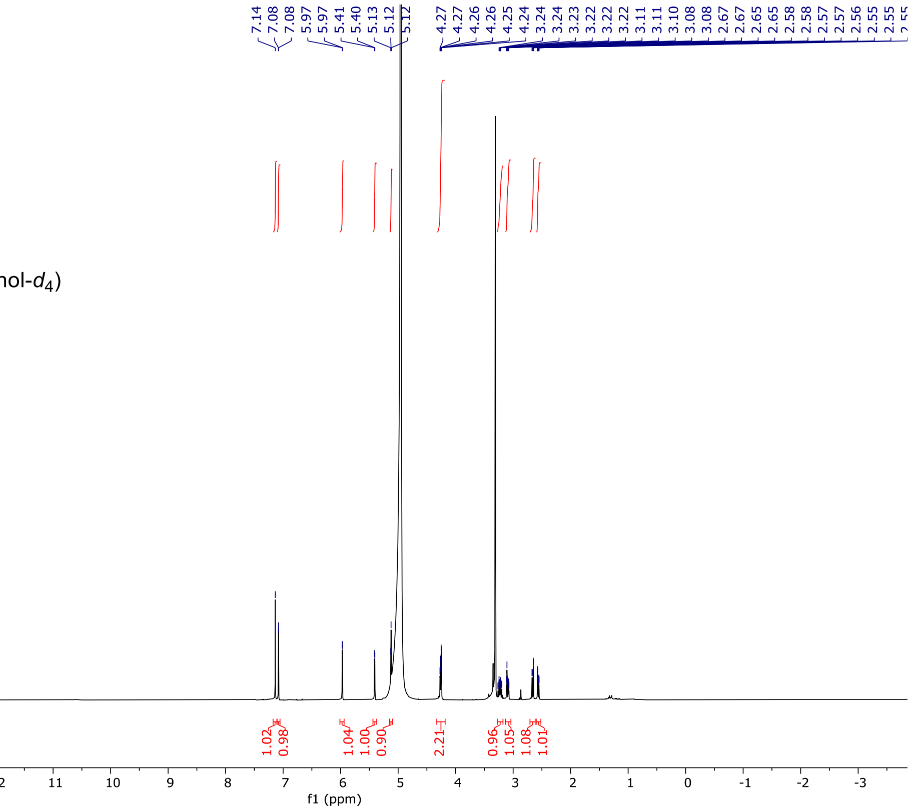

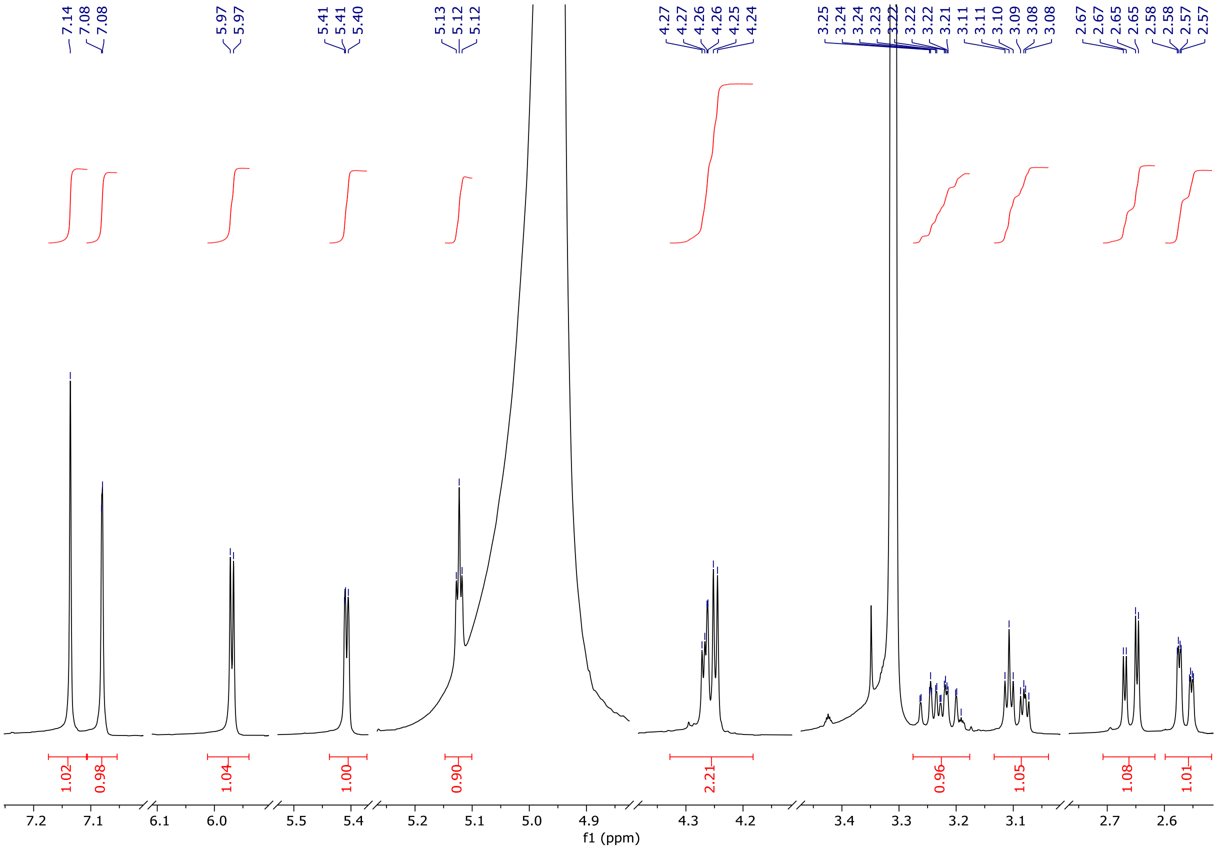

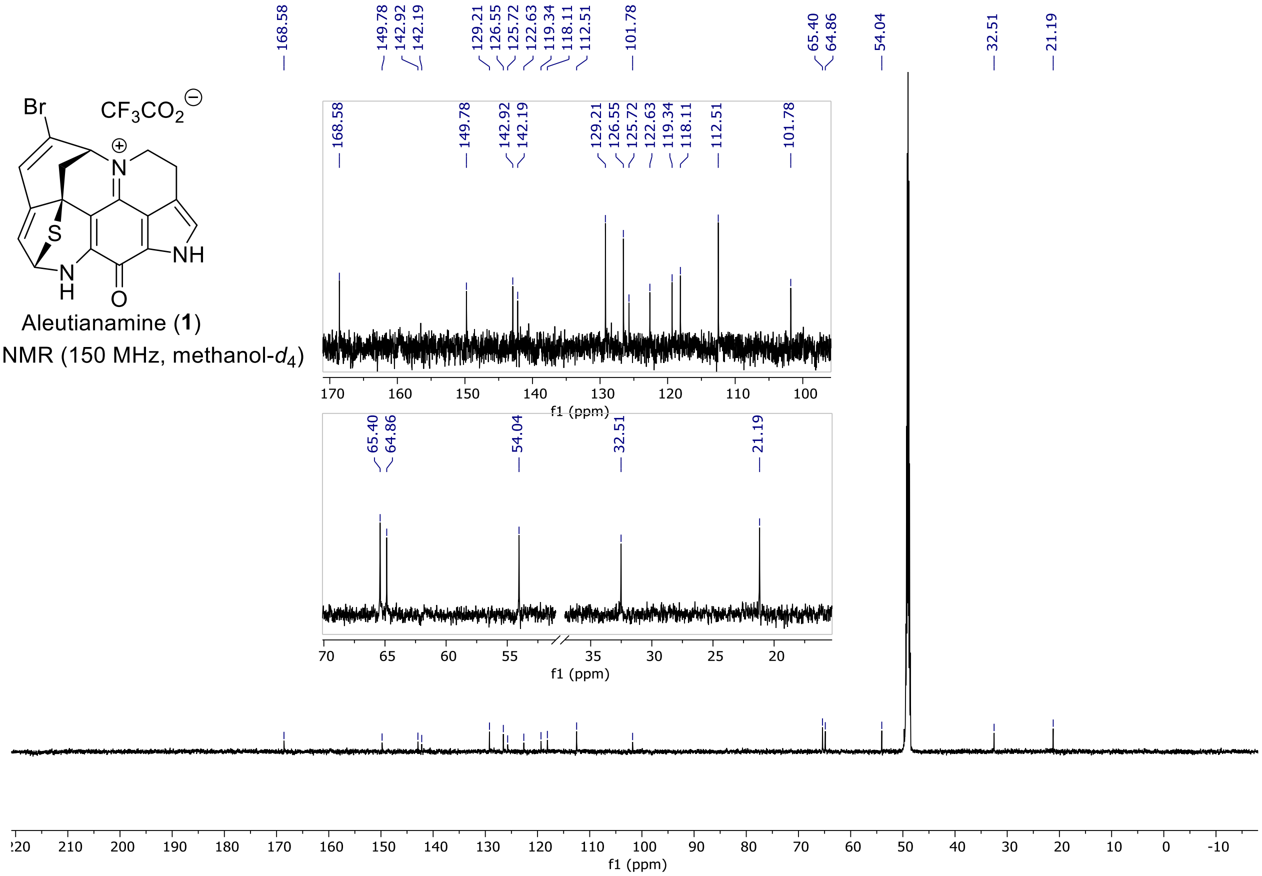

Aleutianamine (**1**)

$^{13}C\{^1H\}$ NMR (150 MHz, methanol-d_4)

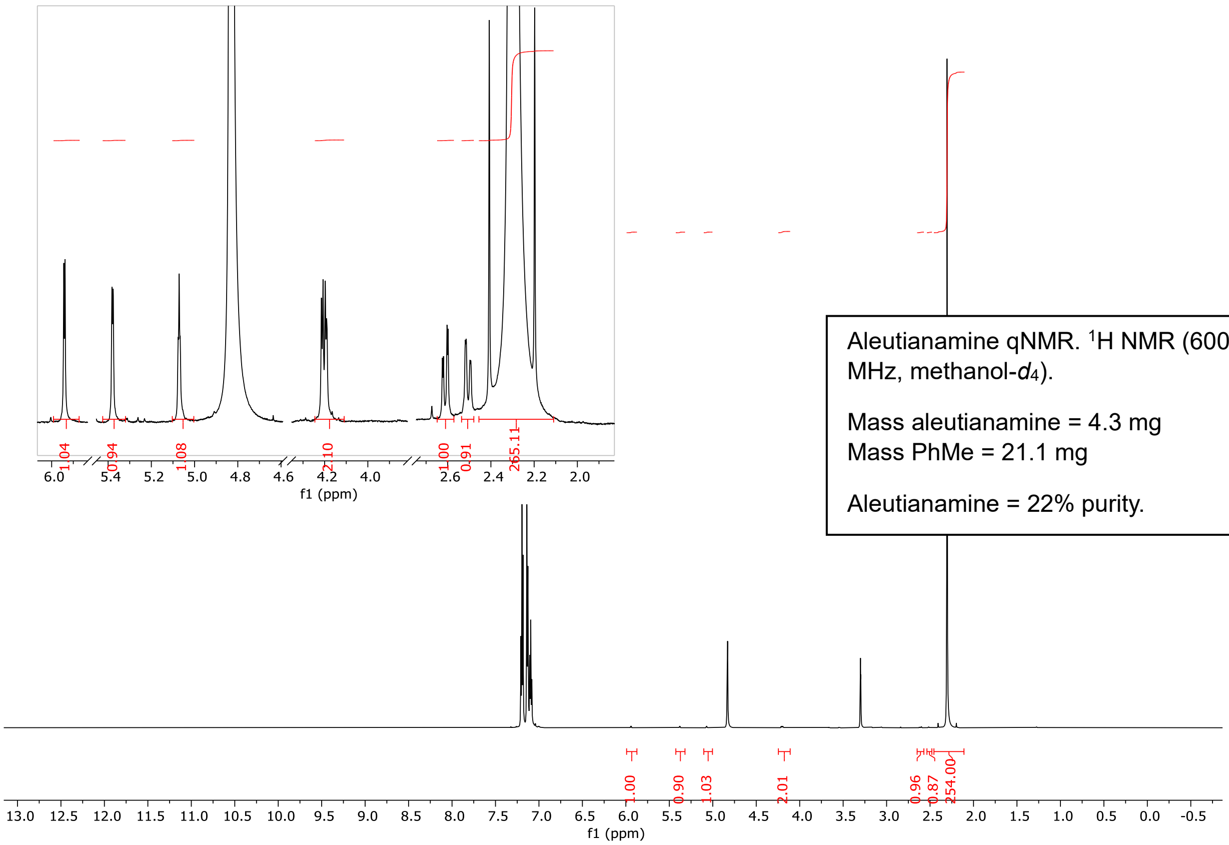

Aleutianamine qNMR. ^1H NMR (600 MHz, methanol-d_4).

Mass aleutianamine = 4.3 mg
Mass PhMe = 21.1 mg

Aleutianamine = 22% purity.

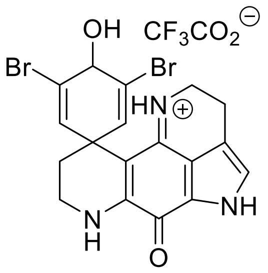

3-DHDC (**6**)

^{1}H NMR (600 MHz, methanol-d_4)

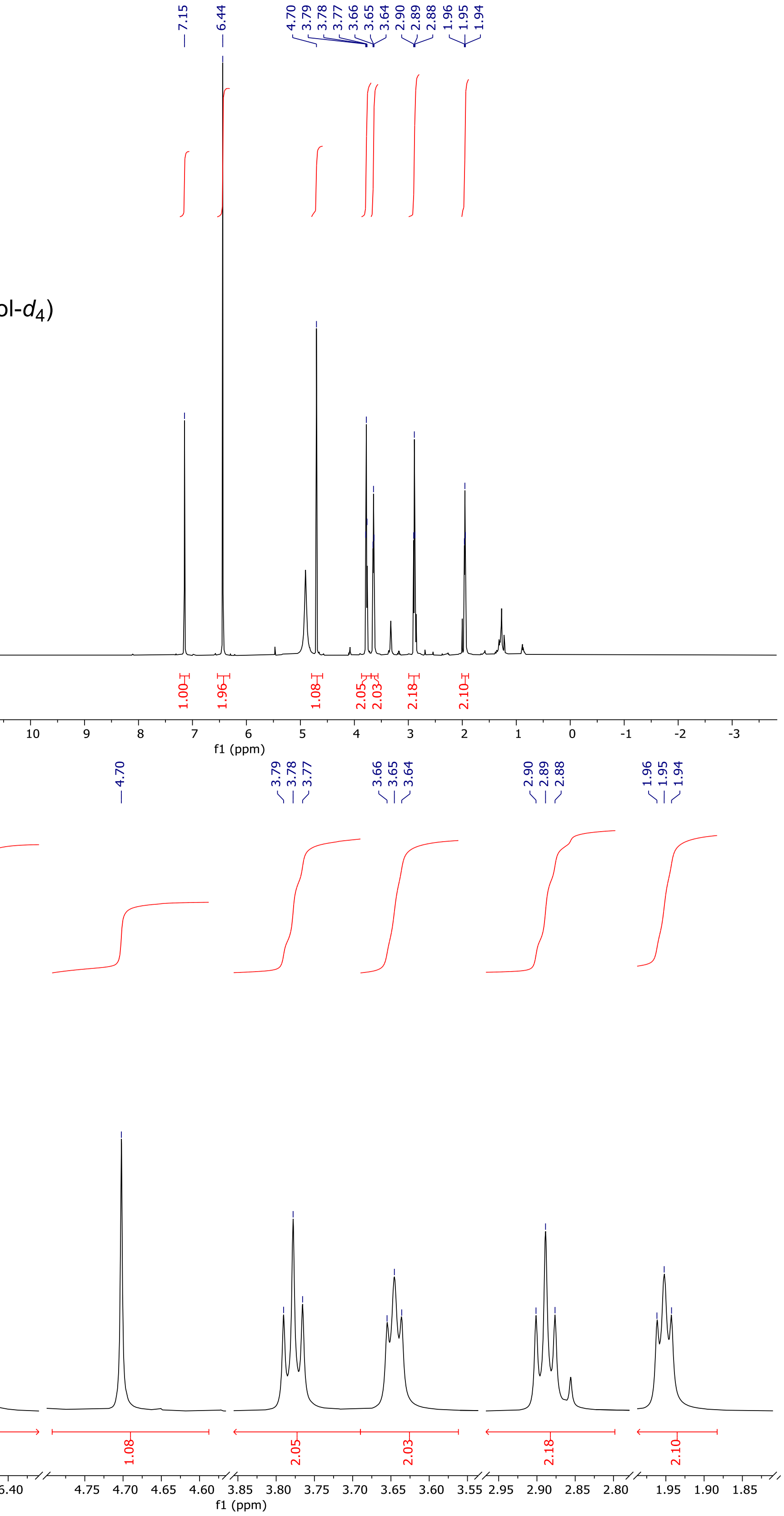

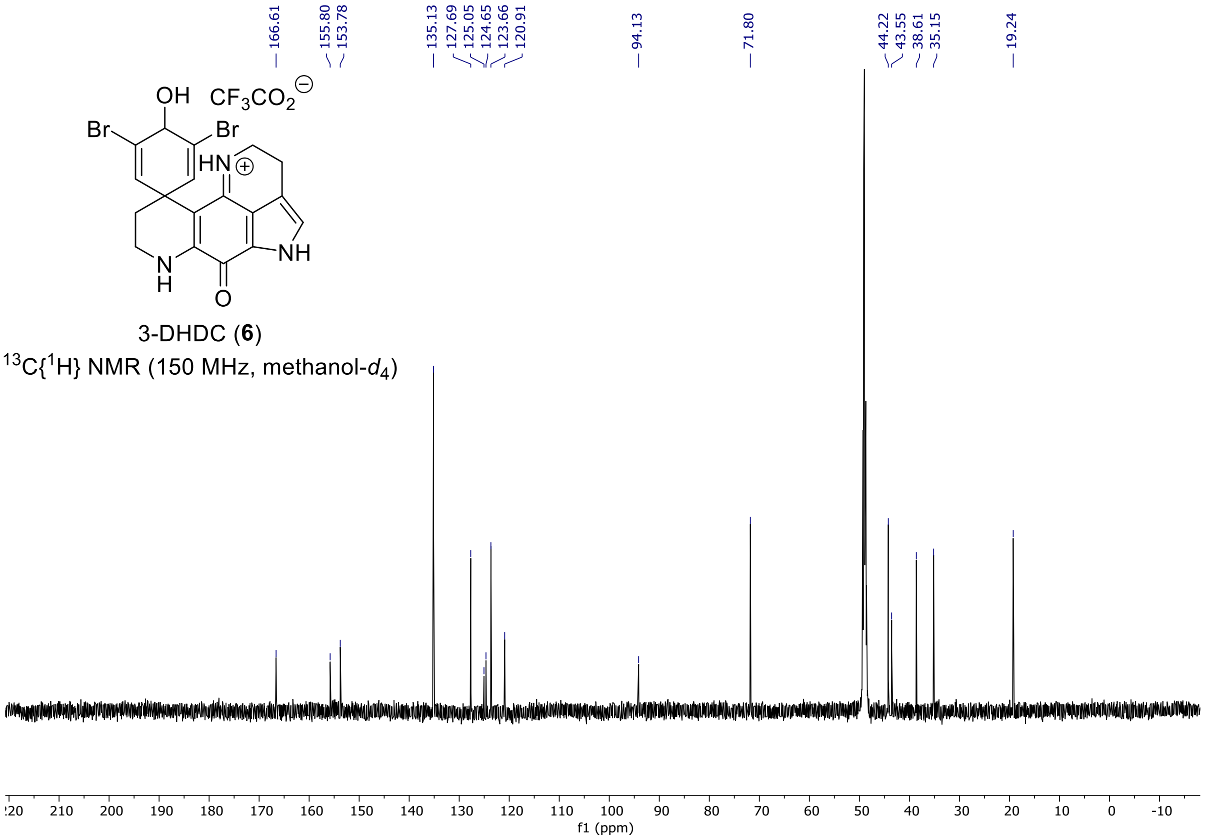

3-DHDC (**6**)

$^{13}C\{^{1}H\}$ NMR (150 MHz, methanol-d_4)

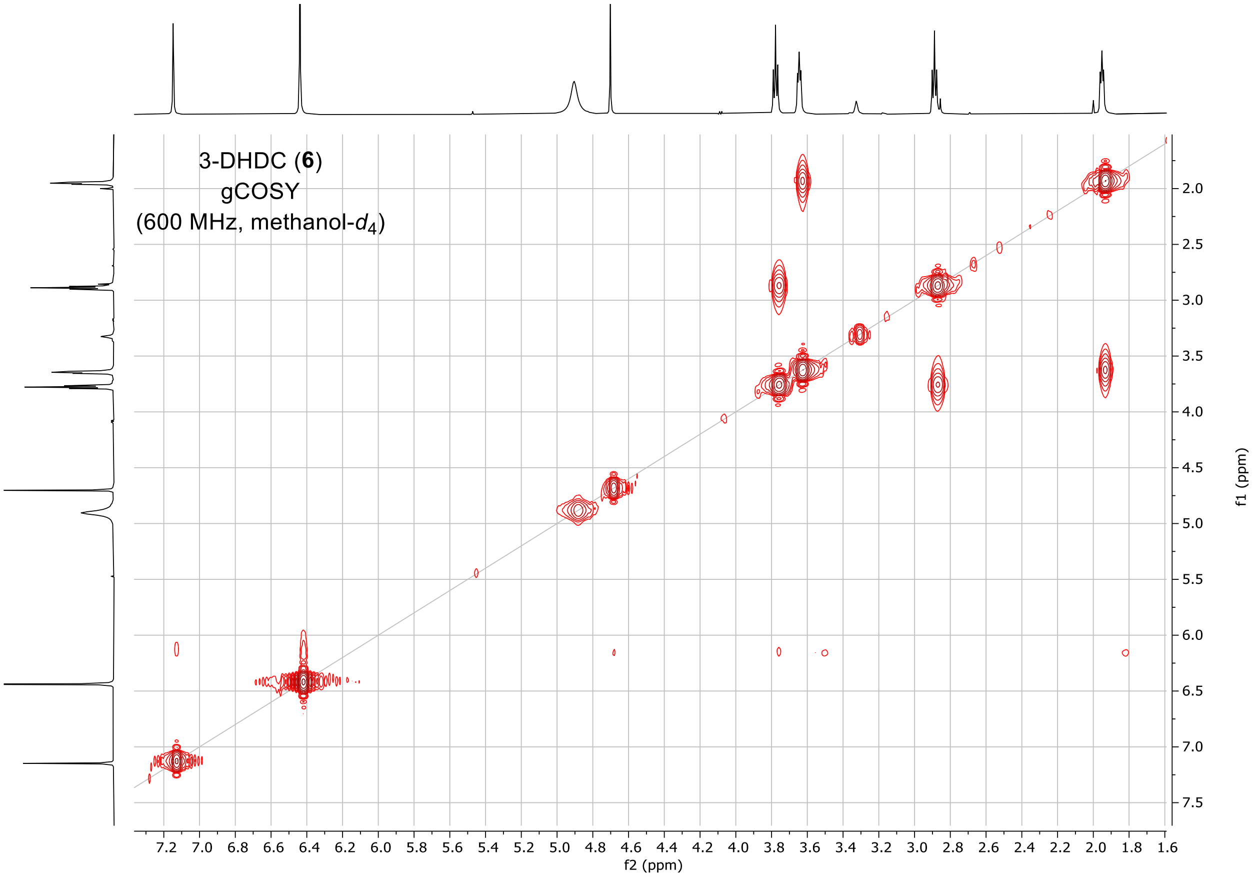

3-DHDC (**6**)
gCOSY
(600 MHz, methanol-d_4)

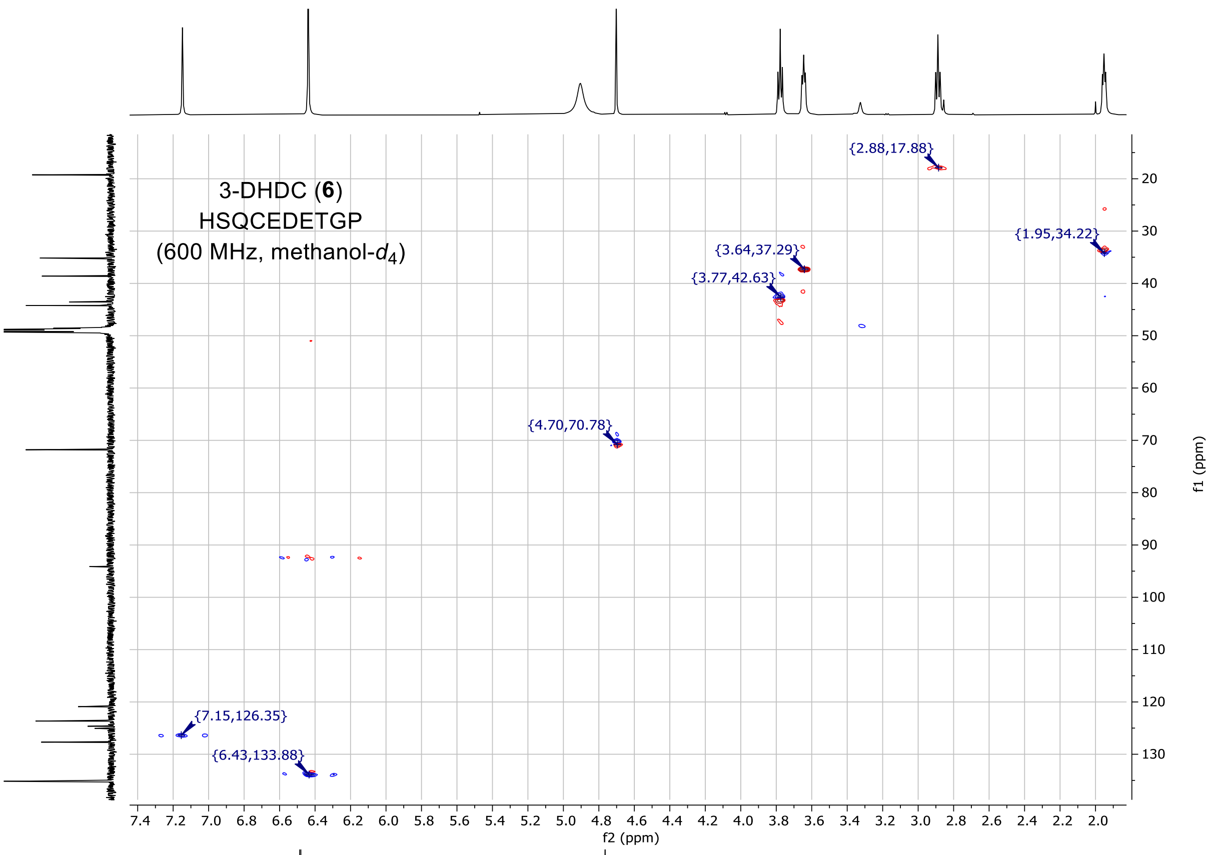
3-DHDC (6)
HSQCEDETGP
(600 MHz, methanol-d4)
{2.88,17.88}
{1.95,34.22}
{3.64,37.29}
{3.77,42.63}
{4.70,70.78}
{7.15,126.35}
{6.43,133.88}
f2 (ppm)
f1 (ppm)

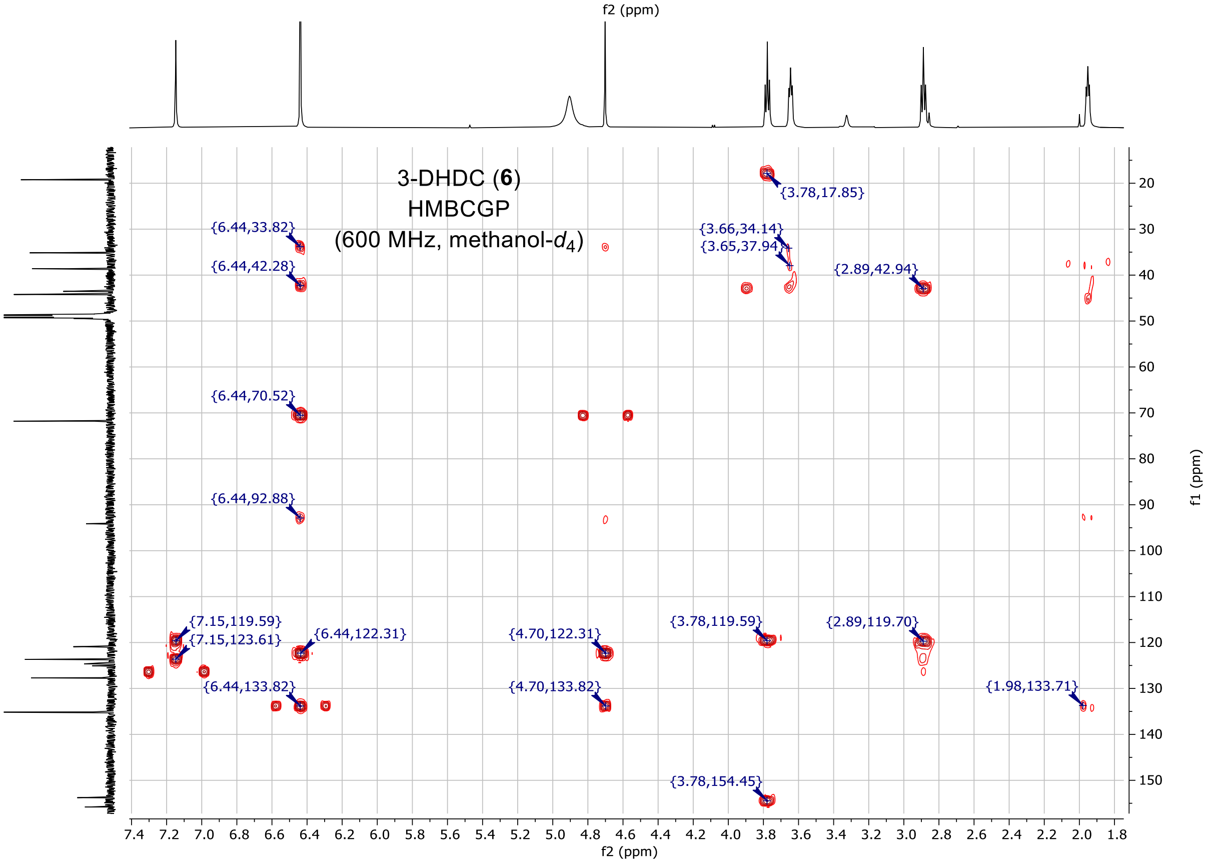
3-DHDC (6)
HMBCGP
(600 MHz, methanol-d4)
{3.78,17.85}
{6.44,33.82}
{3.66,34.14}
{3.65,37.94}
{6.44,42.28}
{2.89,42.94}
{6.44,70.52}
{6.44,92.88}
{7.15,119.59}
{7.15,123.61}
{6.44,122.31}
{4.70,122.31}
{3.78,119.59}
{2.89,119.70}
{6.44,133.82}
{4.70,133.82}
{1.98,133.71}
{3.78,154.45}
f2 (ppm)
f1 (ppm)

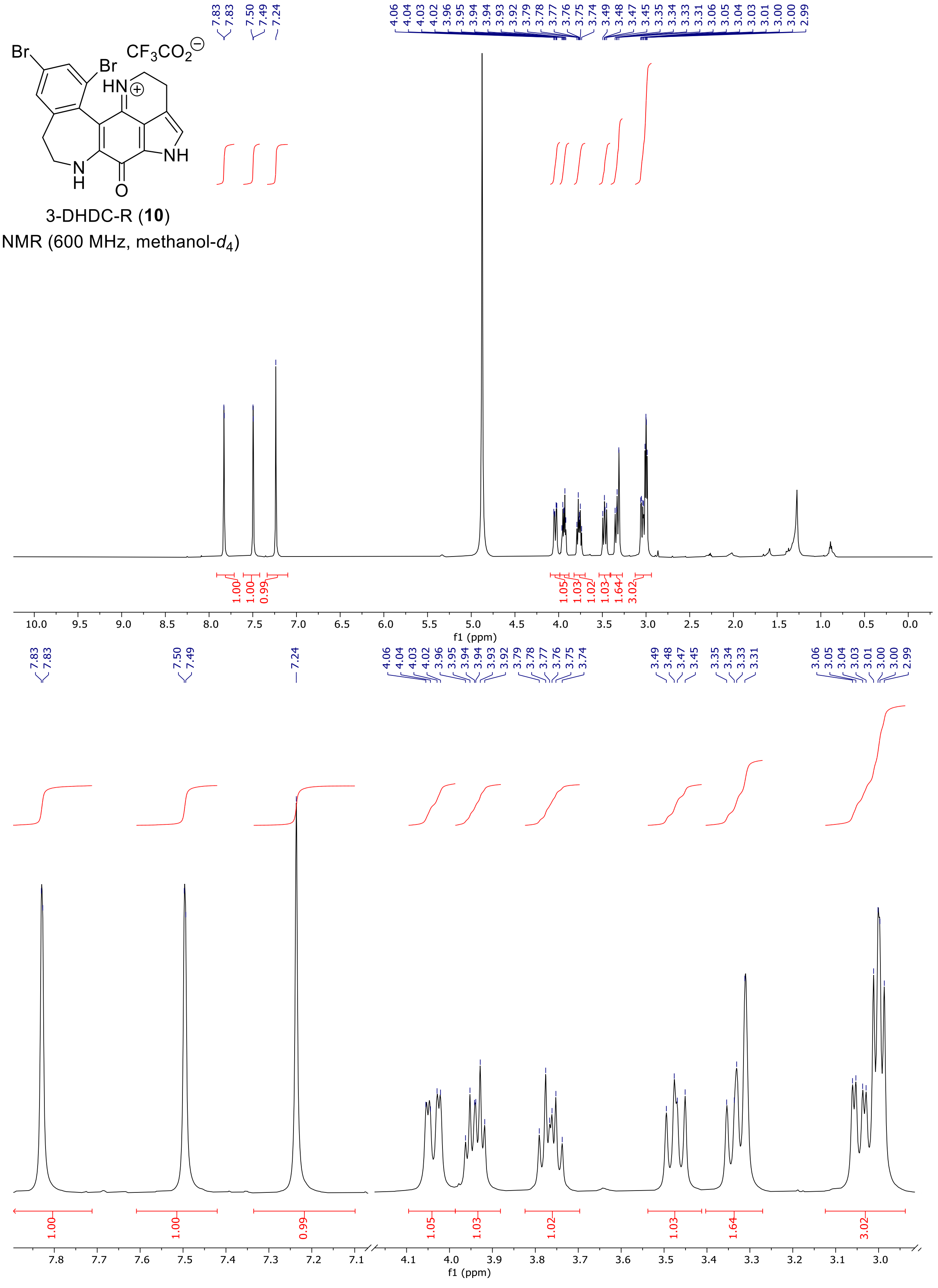

3-DHDC-R (**10**)

^{1}H NMR (600 MHz, methanol-d_4)

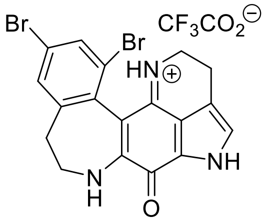

3-DHDC-R (**10**)
$^{13}C\{^{1}H\}$ NMR (600 MHz, methanol-d_4)

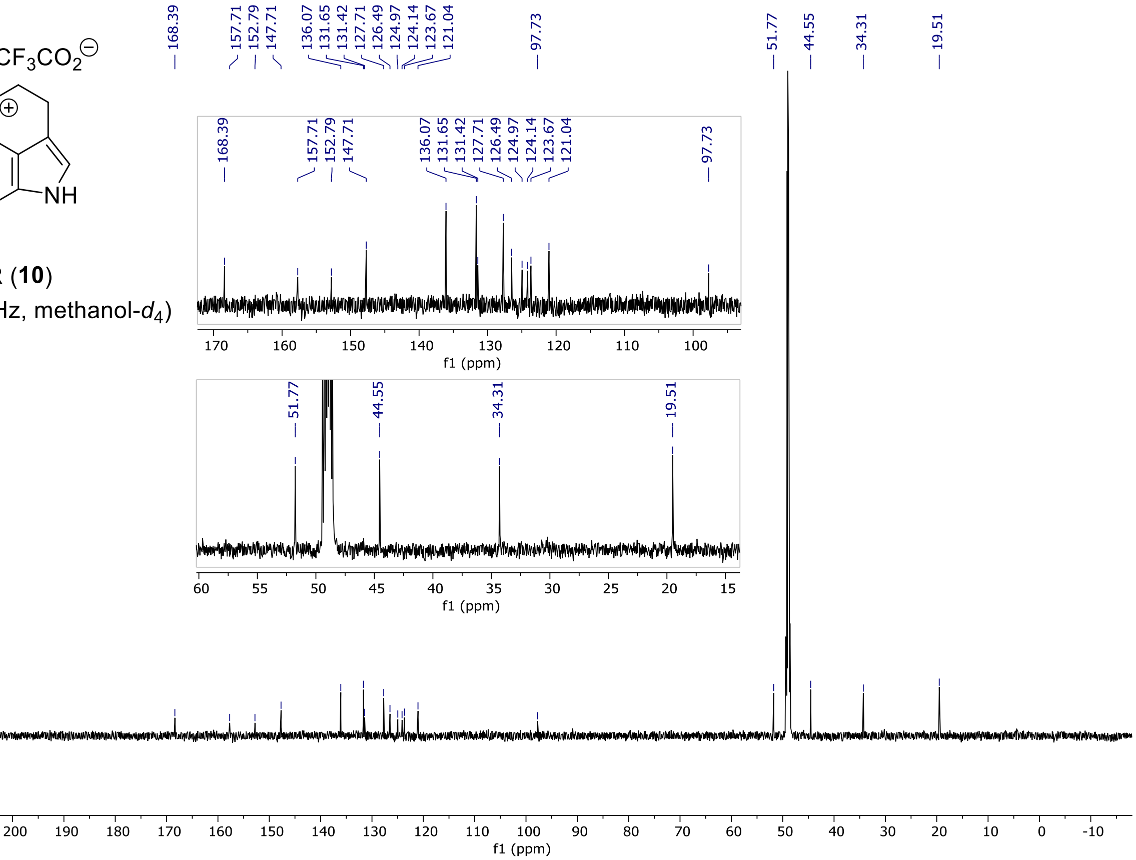

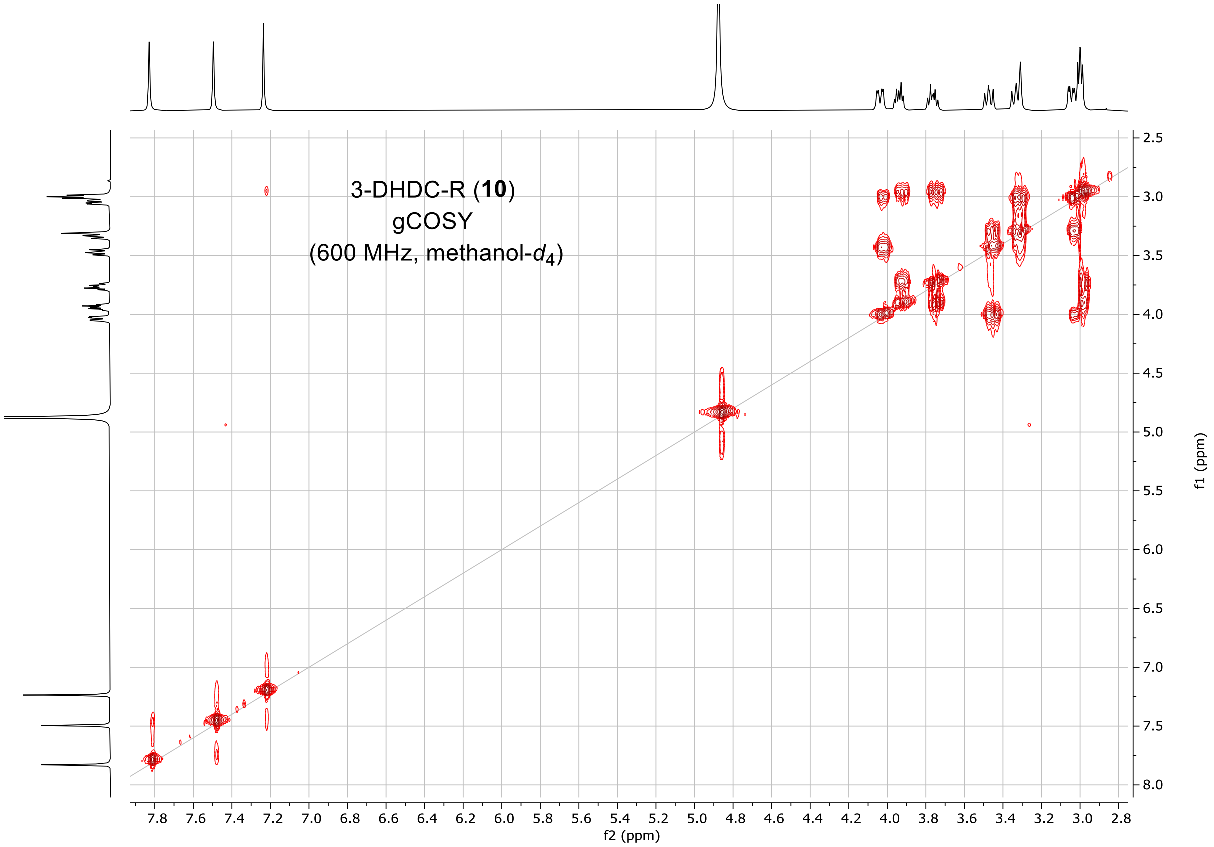

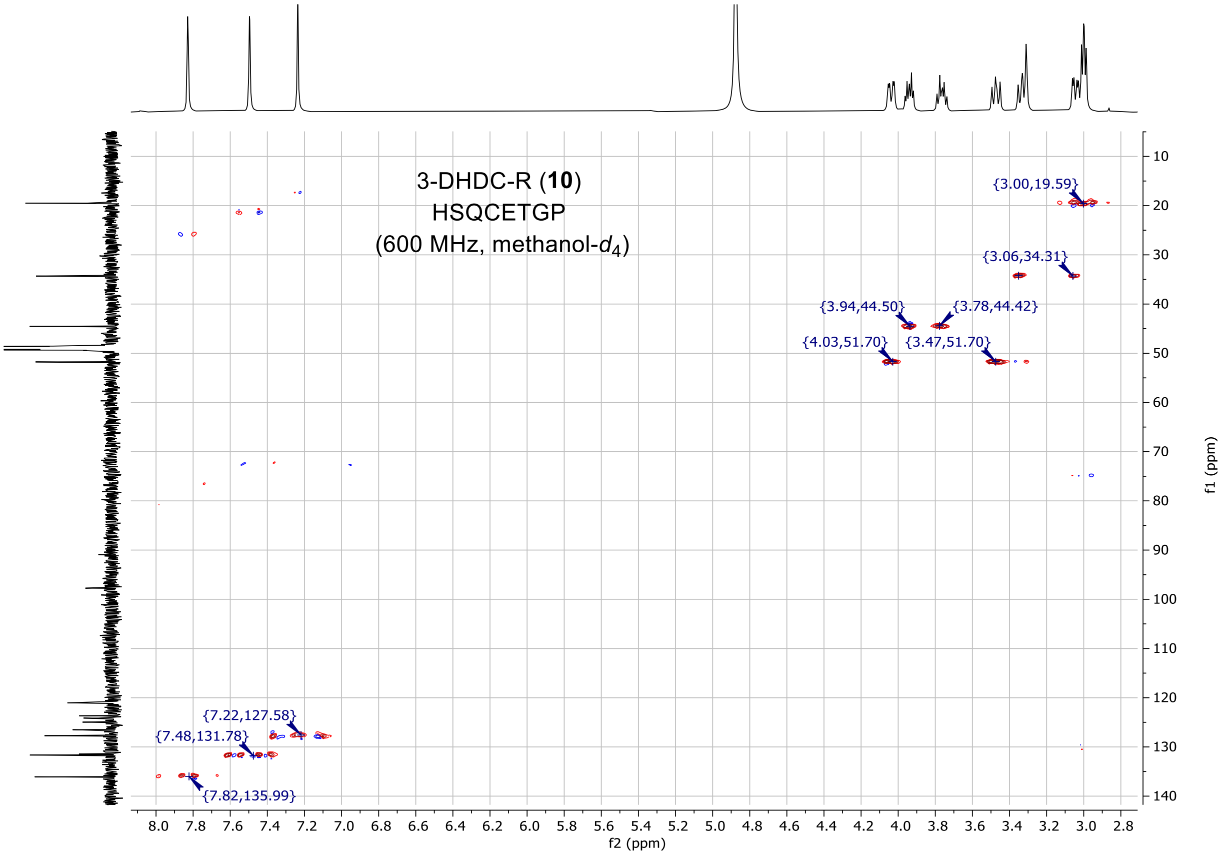
3-DHDC-R (**10**)
HSQCETGP
(600 MHz, methanol-d_4)
{3.00,19.59}
{3.06,34.31}
{3.94,44.50}
{3.78,44.42}
{4.03,51.70}
{3.47,51.70}
{7.22,127.58}
{7.48,131.78}
{7.82,135.99}
f2 (ppm)
f1 (ppm)

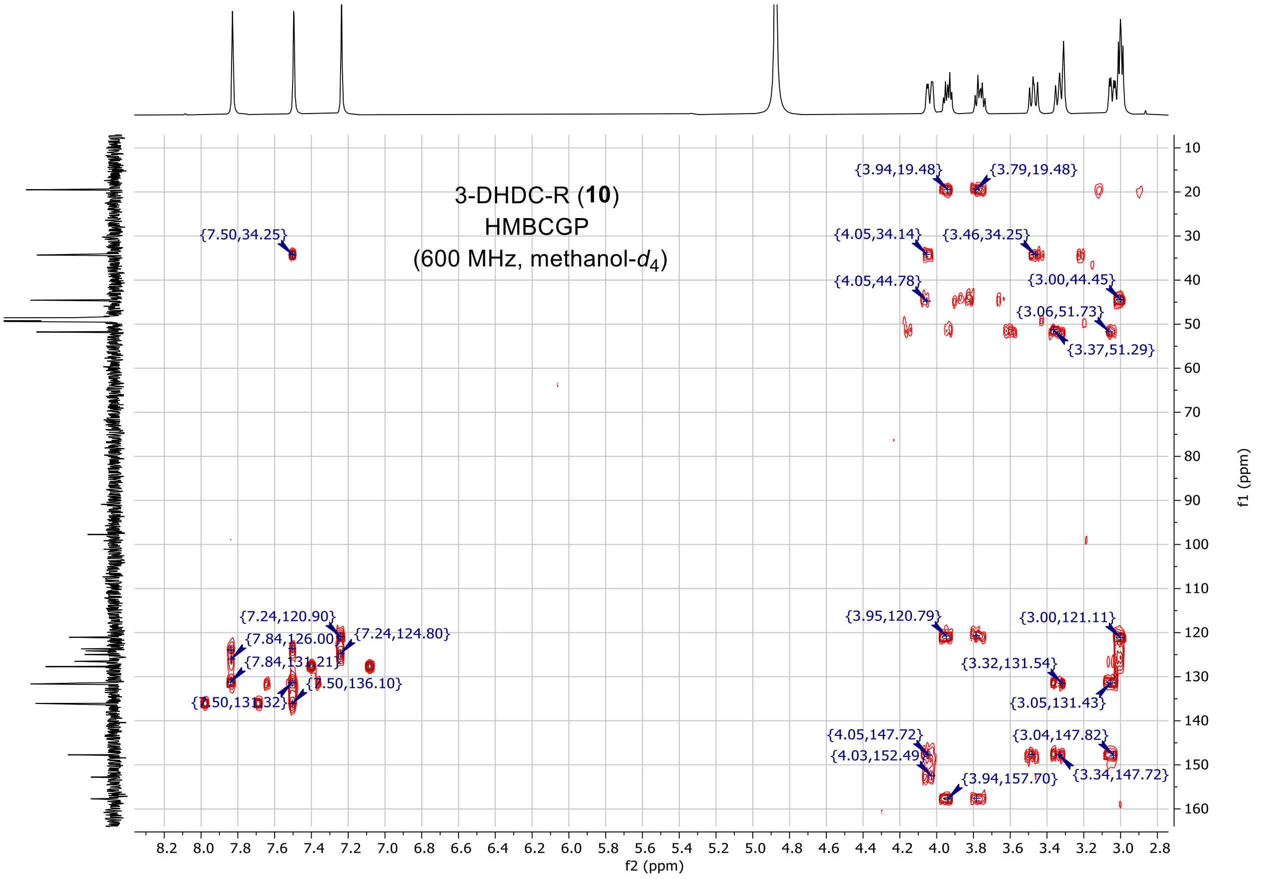
3-DHDC-R (**10**)
HMBCGP
(600 MHz, methanol-d_4)
{3.94,19.48}
{3.79,19.48}
{7.50,34.25}
{4.05,34.14}
{3.46,34.25}
{4.05,44.78}
{3.00,44.45}
{3.06,51.73}
{3.37,51.29}
{7.24,120.90}
{7.24,124.80}
{7.84,126.00}
{7.84,131.21}
{7.50,136.10}
{3.95,120.79}
{3.00,121.11}
{3.32,131.54}
{3.05,131.43}
{4.05,147.72}
{3.04,147.82}
{4.03,152.49}
{3.94,157.70}
{3.34,147.72}
f2 (ppm)
f1 (ppm)

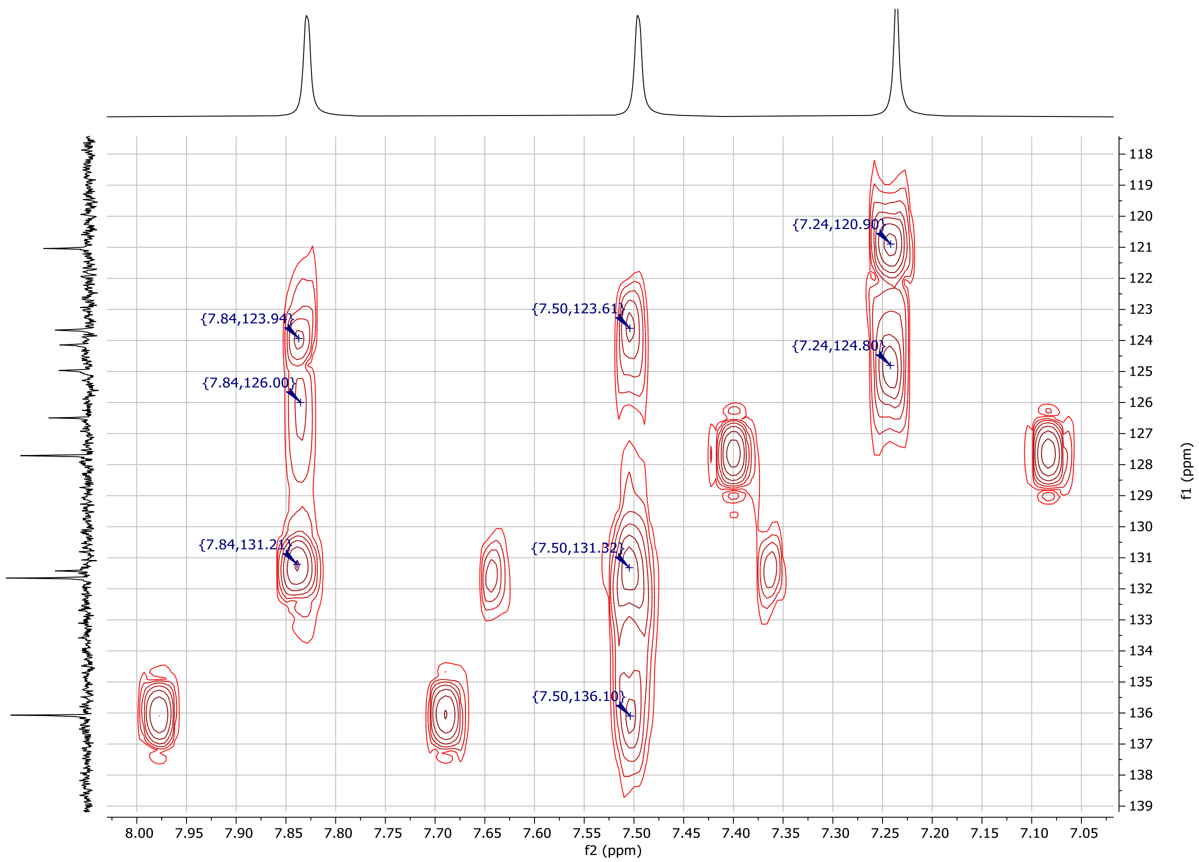

{7.24,120.90}
{7.84,123.94}
{7.50,123.61}
{7.24,124.80}
{7.84,126.00}
{7.84,131.21}
{7.50,131.32}
{7.50,136.10}
f2 (ppm)
f1 (ppm)

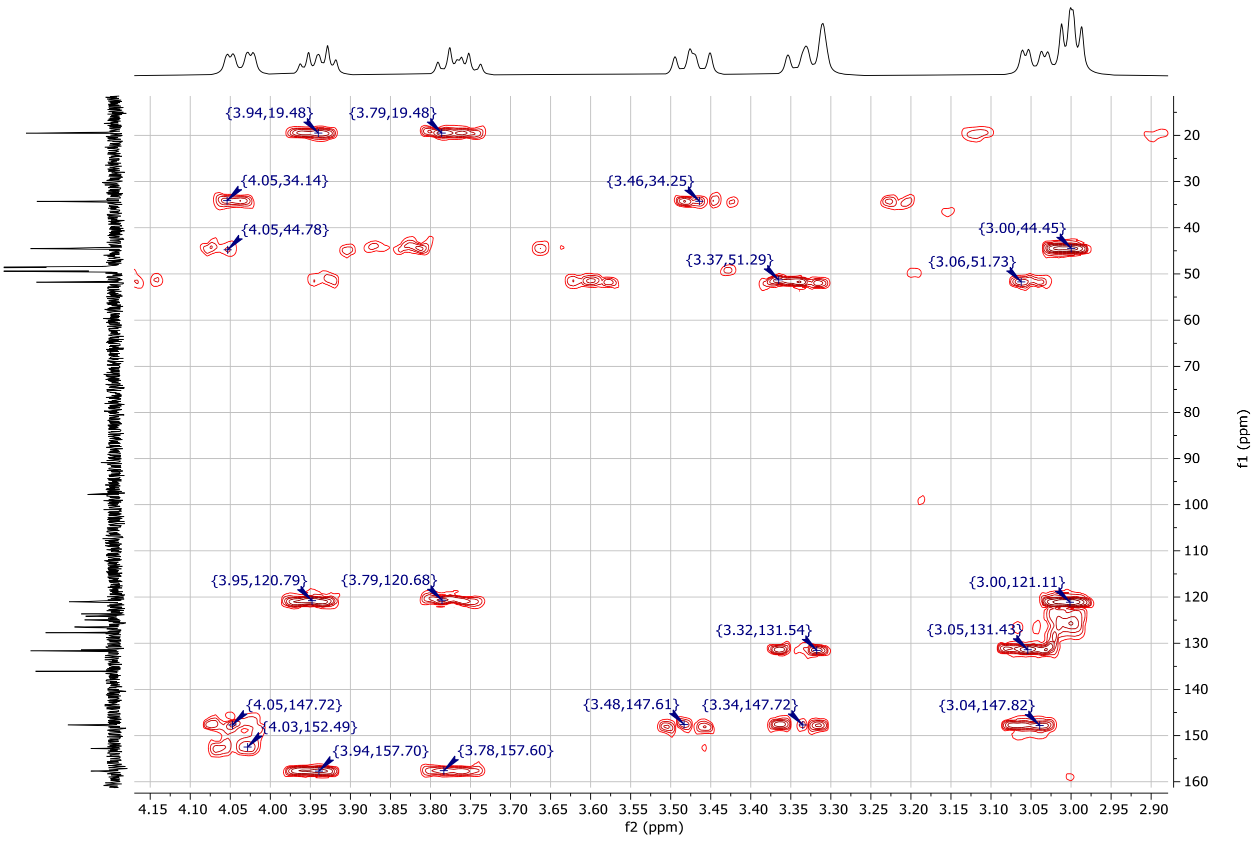

{3.94,19.48}
{3.79,19.48}
{4.05,34.14}
{3.46,34.25}
{4.05,44.78}
{3.00,44.45}
{3.37,51.29}
{3.06,51.73}
{3.95,120.79}
{3.79,120.68}
{3.00,121.11}
{3.32,131.54}
{3.05,131.43}
{4.05,147.72}
{3.48,147.61}
{3.34,147.72}
{3.04,147.82}
{4.03,152.49}
{3.94,157.70}
{3.78,157.60}
f2 (ppm)
f1 (ppm)
